# Antiviral Actions of 25-Hydroxycholesterol in Fish Vary With the Virus-Host Combination

**DOI:** 10.3389/fimmu.2021.581786

**Published:** 2021-02-24

**Authors:** Mikolaj Adamek, Jonathan Davies, Alexander Beck, Lisa Jordan, Anna M. Becker, Miriam Mojzesz, Krzysztof Rakus, Typhaine Rumiac, Bertrand Collet, Graham Brogden, Keith Way, Sven M. Bergmann, Jun Zou, Dieter Steinhagen

**Affiliations:** ^1^ Fish Disease Research Unit, Institute for Parasitology, University of Veterinary Medicine Hannover, Hannover, Germany; ^2^ School of Life Sciences, Keele University, Keele, United Kingdom; ^3^ Institute of Bioprocess Engineering, Friedrich-Alexander-University, Erlangen, Germany; ^4^ Department of Evolutionary Immunology, Institute of Zoology and Biomedical Research, Faculty of Biology, Jagiellonian University, Krakow, Poland; ^5^ Université Paris-Saclay, INRAE, UVSQ, VIM, Jouy-en-Josas, France; ^6^ Department of Physiological Chemistry, University of Veterinary Medicine Hannover, Hannover, Germany; ^7^ Institute for Experimental Virology, TWINCORE, Centre for Experimental and Clinical Infection Research, a joint venture between the Medical School Hannover and the Helmholtz Centre for Infection Research, Hannover, Germany; ^8^ Centre for Environment, Fisheries and Aquaculture Science (CEFAS), Weymouth, United Kingdom; ^9^ Institute of Infectology, Friedrich-Loeffler-Institut (FLI), Greifswald, Germany; ^10^ Key Laboratory of Exploration and Utilization of Aquatic Genetic Resources, Ministry of Education, Shanghai Ocean University, Shanghai, China; ^11^ International Research Center for Marine Biosciences at Shanghai Ocean University, Ministry of Science and Technology, Shanghai, China; ^12^ National Demonstration Center for Experimental Fisheries Science Education, Shanghai Ocean University, Shanghai, China; ^13^ Laboratory for Marine Biology and Biotechnology, Qingdao National Laboratory for Marine Science and Technology, Qingdao, China

**Keywords:** 25-hydroxycholesterol, *cyprinid herpesvirus 3*, *spring viremia of carp virus*, type I interferon, common carp, rainbow trout, Chinook salmon

## Abstract

Cholesterol is essential for building and maintaining cell membranes and is critical for several steps in the replication cycle of viruses, especially for enveloped viruses. In mammalian cells virus infections lead to the accumulation of the oxysterol 25-hydroxycholesterol (25HC), an antiviral factor, which is produced from cholesterol by the cholesterol 25 hydroxylase (CH25H). Antiviral responses based on CH25H are not well studied in fish. Therefore, in the present study putative genes encoding for CH25H were identified and amplified in common carp and rainbow trout cells and an HPLC-MS method was applied for determination of oxysterol concentrations in these cells under virus infection. Our results give some evidence that the activation of CH25H could be a part of the antiviral response against a broad spectrum of viruses infecting fish, in both common carp and rainbow trout cells *in vitro*. Quantification of oxysterols showed that fibroblastic cells are capable of producing 25HC and its metabolite 7α,25diHC. The oxysterol 25HC showed an antiviral activity by blocking the entry of *cyprinid herpesvirus 3* (CyHV-3) into KFC cells, but not *spring viremia of carp virus* (SVCV) or common carp paramyxovirus (Para) in the same cells, or *viral haemorrhagic septicaemia virus* (VHSV) and *infectious pancreatic necrosis virus* (IPNV) into RTG-2 cells. Despite the fact that the CH25H based antiviral response coincides with type I IFN responses, the stimulation of salmonid cells with recombinant type I IFN proteins from rainbow trout could not induce *ch25h_b* gene expression. This provided further evidence, that the CH25H-response is not type I IFN dependent. Interestingly, the susceptibility of CyHV-3 to 25HC is counteracted by a downregulation of the expression of the *ch25h_b* gene in carp fibroblasts during CyHV-3 infection. This shows a unique interplay between oxysterol based immune responses and immunomodulatory abilities of certain viruses.

## Introduction

Cholesterol is an essential lipid for building and maintaining cell membranes and is one of the main components of lipid rafts. These rafts are nanoscale cell membrane assemblies rich in cholesterol, glycosphingolipids, and aggregating proteins, which are important for a range of functions including signaling *via* transmembrane receptors, protein trafficking, and sorting (1). Furthermore, cholesterol also plays a critical role in modulating membrane fluidity. Cholesterol biosynthesis is a product of the mevalonate pathway. This complex reaction cascade requires the activity of enzymes such as 3-hydroxy-3-methylglutaryl coenzyme A (HMG-CoA) synthase and reductase, mevalonate kinase and farnesyl diphosphate synthase (FDPS). The levels of cholesterol are also regulated by enzymatic oxidation, creating oxysterols with a broad range of metabolic and immunological functions (2).

As in mammalian cells, cholesterol is one of the most abundant lipids in fish cells, especially in cholesterol-rich microdomains (rafts) of the plasma membrane, underlining the high similarity of mammalian and fish cells (3, 4). In mammalian cells, cholesterol/lipid rafts are critical for several steps in the viral replication cycle. The distribution of cholesterol in cell membranes was shown to affect entry, replication, and budding of various viruses, especially of enveloped viruses present in mammals and fish (3, 5). In previous research we noted that the reduction of the cholesterol *in vitro* content of the cell membrane severely stunted the entry of the carp pathogenic *cyprinid herpesvirus 3* (CyHV-3) into host cells, but it did not influence the replication of this virus (3). In mammalian cells, the importance of the sterol metabolic network for immune responses is well established (6), and a suppression of cholesterol synthesis is discussed as an important antiviral mechanism of type I interferons (IFN1) (7). This could be mediated by the binding of viperin (virus inhibitory protein, endoplasmic reticulum associated, IFN-inducible) to the farnesyl diphosphate synthase and by this presumably modifying or blocking the FDPS activity (8). In addition, during the antiviral response of mammalian cells, cholesterol is metabolized to the oxysterol 25-hydroxycholesterol (25HC), a soluble antiviral factor. 25HC is generated from cholesterol by activation of cholesterol 25-hydroxylase (CH25H) and is subsequently metabolized to 7α,25di-hydroxycholesterol by 25-hydroxycholesterol 7-alpha-hydroxylase (cytochrome P450 family 7 subfamily B member 1—CYP7B1). This pathway is activated during immune responses (4).

25-Hydroxycholesterol has surprisingly broad antiviral (9) and anticancer effects (10) in mammalian cells and targets early stages of virus infections by blocking virus entry into the cell. The mechanisms of stopping virus entry involve changes to the charge of the cell membrane, sequestration of available cholesterol required for the attachment of the virus to the cell, or disruption of the viral envelope (9). 25HC can directly influence virus replication by interacting with viral proteins and stopping them from forming complexes required for virus replication (11). Furthermore, 25HC stimulates the expression of miRNA inducing antiviral activity (12). The replication of un-enveloped viruses is suppressed by targeting members of the oxysterol binding protein (OSBP) family I at the Golgi apparatus in picornavirus infected cells (13).

In fish, alterations of cholesterol synthesis and metabolism in the context of viral infections are scarcely explored. The sterol pathway was affected in the brain of survivors and carriers of *infectious hematopoietic necrosis virus* (IHNV) in sockeye salmon (*Oncorhynchus nerka*), and a prolonged downregulation of the genes encoding for enzymes involved in cholesterol synthesis was noticed. This could suggest that after an infection with this rhabdovirus, there is a continued restriction of cholesterol synthesis in fish surviving the infection (14). An antiviral response based on 25HC also seems to be present in fish. For example: the *ch25h-like* gene was regulated during a PRV-1 infection in Atlantic salmon (*Salmo salar*) (15). Furthermore, in zebrafish (*Danio rerio*) several paralogues of human cholesterol 25-hydroxylase have been described with the mRNA expression of one paralogue *ch25h_b* being highly inducible by viral infections (16). An over-expression of *ch25h_b* had an antiviral activity against *spring viremia of carp virus* (SVCV) (16). This suggests that a cholesterol 25-hydroxylase-based immune response is highly conserved in vertebrates and could play a profound role in their antiviral responses. Additionally, the authors of this study concluded that the activation of *ch25h* was not dependent on IFN1 (16) and thus contributed to the dispute about the IFN dependence of this response taking place in higher vertebrates (17). In orange-spotted grouper (*Epinephelus coioides*), the overexpression of cholesterol 25-hydroxylase decreased both replication and entry of *Singapore grouper iridovirus* (SGIV) and *red-spotted grouper nervous necrosis virus* (RGNNV) in a grouper spleen cell line (18). One of the proposed indirect mechanisms by which 25HC can affect the viral replication in fish cells is its binding to the monomeric c-reactive protein (CRP) isoforms and boosting of the antiviral activities of CRP (19) inhibiting autophagic processes (20).

Here we study *ch25h_b* activation and its involvement in the antiviral response of cells against a very broad spectrum of viruses in common carp (*Cyprinus carpio*), rainbow trout (*Oncorhynchus mykiss*), and Chinook salmon (*Oncorhynchus tshawytscha*) *in vitro*. For this, we analyzed, whether fibroblastic cells of carp are able to produce 25HC and its metabolite 7α,25diHC. We subsequently studied whether 25HC has an antiviral activity and blocks the entry of the fish-pathogenic viruses CyHV-3, SVCV, common carp paramyxovirus (Para), *viral haemorrhagic septicaemia virus* (VHSV), and *infectious pancreatic necrosis virus* (IPNV). We selected these viruses not only because of their importance in aquaculture and association with significant economic losses, but also because of their diversity in entry mechanism. As mentioned above, CyHV-3 most likely uses lipid rafts for entry into carp cells (3). Both rhabdoviruses, VHSV and SVCV, most likely utilize clathrin-mediated endocytosis and macropinocytosis dependent on low-pH (21, 22). The un-enveloped IPNV uses mainly micropinocytosis rather than clathrin-mediated or lipid raft/caveolin-mediated endocytosis (23, 24). While the entry mechanism of fish paramyxoviruses has not been studied the results obtained for paramyxoviruses of higher vertebrates suggest cholesterol-rich lipid raft involvement (25). We hypothesize that the utilization of divergent entry pathways combined with significant differences in the induction of antiviral responses by these viruses could lead to a different effect of 25HC on their entry. During this study we established further evidence of *ch25h_b* antiviral activity independent from type I IFN activation. We also show that the successful replication of CyHV-3 may depend on blocking the antiviral responses leading to changes in cholesterol or oxysterols synthesis.

## Material and Methods

### Cells

Koi fin cells (KFC), koi fin-1 (KF-1), and common carp brain (CCB) permanent fibroblast-like cell lines from common carp (26–28), were provided by the Collection of Cell Lines in Veterinary Medicine (CCLV), at Friedrich-Loeffler-Institut (FLI), Federal Research Institute for Animal Health, Greifswald—Insel Riems, Germany. The cells were cultured in minimum essential medium (MEM) with Earle’s salts (Sigma, Germany) supplemented with non-essential amino acids (NEAA; Sigma), 10% fetal calf serum (FCS, Sigma), 10 IU/ml penicillin and 100 mg/ml streptomycin (Sigma). Cultures were incubated at 20°C in a humidified atmosphere containing 2% CO_2_. The KFC cells were plated into 12-well plates, 96-well plates or T75 culture flasks, while KF-1 and CCB cells were placed into T25 culture flasks or 96-well plates. The cells were grown for 24h until reaching 95% confluence before treatment or infection. Rainbow trout gonad 2 (RTG-2) cells, a permanent fibroblast-like cell line from rainbow trout (29) were also provided by CCLV at FLI. The cells were cultured in MEM with Earle’s salts supplemented with 10% FCS, 10 UI/ml penicillin, and 100 mg/ml streptomycin. Cultures were incubated at 20°C in a humidified atmosphere containing 2% CO_2_. Similar to KFC cells, RTG-2 cells were plated into 12-well plates and grown for 24h and utilized upon reaching 95% confluence before treatment or infection. Chinook salmon (*Oncorhynchus tshawytscha*) CHSE-EC is a clonal cell line derived from CHSE-214 (ATCC CRL1681), which constitutively expressed mEGFP and nCas9n. The *stat1a1/2* knock-out CHSE-GS1A cells with point deletions of *stat1a1* and *stat1a2* were derived from CHSE-EC (30). Both cell lines were developed and provided by the French National Research Institute for Agriculture, Food, and Environment (INRAE). The cells were cultured in GMEM (custom made by Eurobio) with L-glutamine supplemented with 10% FCS, 10 IU/ml penicillin, 100 mg/ml streptomycin, 500 μg/ml G418, and 30 μg/ml hygromycin. Cells were incubated at 20°C in a humidified atmosphere containing 5% CO_2_. Similar to other cells CHSE-EC and CHSE-GS1A cells were plated into 12-well plates and grown for 24h and utilized upon reaching 95% confluency.

### Viruses

The cyprinid herpesvirus 1 (CyHV-1, isolate CyHV-1UK) and common carp iridovirus (CCIV isolate 4BG) were provided by K. Way from the Centre for Environment, Fisheries, and Aquaculture Science (CEFAS), Weymouth, United Kingdom (27, 31, 32). CyHV-3 (Taiwan isolate) was provided by S.M. Bergmann from the FLI. Common carp paramyxovirus (isolate 1551, referred hereafter as “Para”), common carp orthomyxovirus (isolate 1617, referred hereafter as “Ortho”), and common carp birna/reovirus (isolate 1620, referred hereafter as “Birna”) were isolated from common carp gills in our laboratory as described by Neukirch and Kunz (33). *Infectious pancreatic necrosis virus* (IPNV serotype Sp) was provided by W. Ahne, University of Munich, Munich, Germany, *spring viremia of carp virus* (SVCV, isolate 56–70) and *viral hemorrhagic septicemia virus* (VHSV, isolate 07.71) were provided by P. de Kinkelin, INRA, Jouy-en-Josas, France, *chum salmon reovirus* (CSV isolate 017.94) was provided by S. LaPatra, Clear Springs Foods, Buhl, USA as described previously (34). The CyHV-1, CCIV, CyHV-3, SVCV, Para, Ortho, and Birna viruses were prepared and titrated using CCB cells. IPNV, CSV, and VHSV isolate 07.71 were replicated in RTG-2 cells. The susceptibility of the KFC and RTG-2 cells to all viruses at 20°C was tested by observation of cytopathic effects and an increase of viral RNA measured by RT-qPCR. For the CHSE-EC and CHSE-GS1A cell lines rVHSV-Tomato derived from the hypervirulent VHSV 23–75 French strain was used. rVHSV-Tomato allowed for better monitoring of viral replication in the cells non-destructively by measuring the fluorescence. This permitted a tight control of the sampling regime to get optimal kinetics of infection/induction of host genes (35, 36). The virus was propagated in epithelioma papulosum cyprini (EPC) cells. The virus work was performed under BSL1 (t2) condition as prescribed by the Technical Rules for Biological Agents, Classification of viruses in risk groups (Technische Regeln für Biologische Arbeitsstoffe, Einstufung von Viren in Risikogruppen—TRBA 462, Germany). Under BSL1 (t2) condition safety measures comparable to the BSL2 are required to minimize the escape of animal viruses into the external environment or other working areas.

### Poly I:C, B-DNA, Z-DNA, and Plasmid Transfection Assays

The cells were transfected using the LyoVec (InvivoGen, France) reagent according to the manufacturer’s instructions with 1 µg/ml of polyinosinic polycytidylic acid (poly I:C synthetic double stranded RNA; InvivoGen, catalogue # tlrl-pic), 1 µg/ml poly(deoxyadenylic-deoxythymidylic) acid sodium salt (synthetic double stranded B-DNA; InvivoGen, catalogue # tlrl-patn) or 1 µg/ml poly(deoxyguanylic-deoxycytidylic) acid sodium salt (synthetic double stranded Z-DNA; InvivoGen, catalogue # tlrl-patn), and medium without FCS as described previously (37). The untransfected controls were treated only with medium containing LyoVec. Replicates (treated with n = 3 separate preparations of the ligands) were collected at 2, 8, 36, and 96h post-transfection. For CHSE-EC and CHSE-GS1A cells the samples (n = 5) were collected at 2, 8, 30, and 96h post poly I:C transfection. CHSE-EC and CHSE-GS1A cells were also transfected with plasmids expressing Atlantic salmon IFN a2 (GenBank ID: AY216595) or IFN γ (GenBank ID: AY795563), the plasmid construction and transfection process was performed as described previously (38). Replicates (n = 3) were harvested 2 or 3 days after transfection. The cells were collected into 1 ml Tri-Reagent (Sigma) and stored as detailed above.

### Virus Infections

The KFC cell cultures were infected with CyHV-1, CCIV, CyHV-3, SVCV, Para, Ortho, and Birna, IPNV, CSV, and VHSV isolate 07.71 at a MOI of 10. RTG-2 cell cultures were infected with IPNV, VHSV isolate 07.71 at a MOI of 10. The CHSE-EC and CHSE-GS1A cell lines were infected with rVHSV-tomato at a MOI of 1. Replicates (n = 3) from KFC and RTG-2 cells were collected at 2, 9, 30, 70 h post-infection (hpi) by lysing the cells in 1 ml Tri-Reagent (Sigma) before being transferred into 1.5 ml reaction tubes and stored at −80°C until RNA extraction. For each of the cell lines a non-infected control was also collected at each of the time points.

### Recombinant Rainbow Trout Type I Interferon Treatment

Recombinant IFN1 from rainbow trout (rtIFN1 or rtIFNa) was prepared as described previously (39). The RTG-2 and KFC cells were stimulated with 100 ng/ml according to the procedure used in earlier studies (39, 40). For analysis, n = 3 replicates were collected after 3, 6, and 24h post-stimulation. The cells were lysed in 1 ml Tri-Reagent (Sigma, Germany) before being transferred into 1.5 ml reaction tubes and stored at −80°C until RNA extraction.

### Virus Entry, Attachment, and Infection Evaluation

Studies on virus entry into KFC and RTG-2 cells were performed as previously described when examining the influence of methyl-beta-cyclodextrin (MβCD) on the entry of CyHV-3 into CCB cells (3). Prior to incubation, MβCD (Sigma, Germany) and 25HC (Cayman Chemical, USA) were dissolved in 100% ethanol (Carl Roth, Germany). The cells were incubated with 10 mmol of MβCD for 30 min in medium without FCS, or with different concentrations of 25HC (1, 10 µM) for 8h. The 25HC was dissolved in the cell culture medium without FCS supplemented with 0.23% of lipid free BSA (Sigma) to prevent adhesion of 25HC to culture vessels. Then, KFC cells treated with MβCD were washed three times with PBS and infected with CyHV-3, SVCV, or Para at a MOI of 1 at 20°C, while the RTG-2 cells were washed three times with PBS and infected with VHSV or IPNV at a MOI of 1 at 20°C. Two hours post-infection at 20°C, the cells were washed three times with medium and then frozen in 1 ml of medium. KFC cells treated with 25HC were washed three times and infected with CyHV-3, SVCV, or Para at a MOI of 0.01 on ice, while the RTG-2 cells were washed and infected with VHSV or IPNV at a MOI of 0.01 on ice. After virus attachment (1 h exposure of cells to virus suspension performed on ice) the cells were washed three times with medium and incubated for 48h at 20°C and then frozen in 1 ml of medium. Afterward, the virus content was evaluated by determining the 50% tissue culture infective dose (TCID_50_) for the respective virus. The CyHV-3 entry experiment was repeated with KF-1 cells at 20°C, additional treatment of the cells with 25HC post-infection was performed. KF-1 cells were pre-treated for 8h with 10 µM of 25HC and infected with MOI of 0.001 for 1h, following inoculation in medium with 10 µM of 25HC. For this experiment the virus content was evaluated in cells and medium after 96h from infection by qPCR and TCID_50_.

Additionally, virus attachment was checked with KFC and RTG-2 cells by repeating the 8h pre-incubation of cells with 25HC and performing the infection on ice for 2h at a MOI of 1. Two hours post-infection, the cells were washed three times with ice cold PBS and then frozen in 100 µl of PBS. After defrosting, cells were centrifuged at 20 000 × g. The supernatant was removed and the cell pellet infected with CyHV-3 was re-suspended in 180 µl of ATL buffer (QIAGEN, Germany) for DNA extraction. Cell pellets infected with SVCV, Para, VHSV, and IPNV were re-suspended in 1 ml of Tri-Reagent (Sigma) for subsequent RNA extraction.

To further evaluate the antiviral effect of the stimulation of cells with 25HC post-virus infection, KFC cells were stimulated with different concentrations of 25HC (5 and 50 µM) for 8h before or 8h after infection with CyHV-3 at a MOI of 1 for 1h. Cells from all treatments (pre-incubated and post-infection incubated with 25HC and non-treated controls) were washed three times prior to infection, 1h after infection and 9h post-infection. During treatment, cells were incubated at 20°C and harvested at 24 or 72h post-infection. At these time points the cells were collected into 1 ml of Tri-Reagent (Sigma) for subsequent RNA extraction.

In order to check the effect of pre-treatment of virus particles with 25HC on the ability of viruses to enter the cells, n = 3 virus suspensions were incubated for 8h with different concentrations of 25HC (1, 10, and 100 µM) or control medium not containing 25HC and thereafter, these virus suspensions were used for infecting KFC or RTG-2 cells at an MOI of 1 as described above. After 2h, the cells were washed three times and frozen in 1 ml medium for determination of virus concentration using the TCID_50_ method.

The zoledronic acid (ZOL) was used to evaluate the influence of FDPS blocking on the susceptibility of KFC cells to the infection with CyHV-3, SVCV, and Para. The KFC cells in 96-well plates (n = 6 replicates each) were pre-treated with 0.1, 1, and 10 µM of zoledronic acid (Sigma) for 24 h, as a control cells with medium not containing the ZOL were used. Then, the susceptibility of the cells for CyHV-3, SVCV, and Para was analyzed by infecting the cells with a serial dilution of the viruses (n = 6 preparation per virus). The viruses were diluted from 1:10^2^ to 1:10^8^ in the medium containing ZOL in above mentioned concentrations and incubated for 7 days, afterward the TCID_50_ was calculated for each of the treatments. For measuring the effect of ZOL on the mRNA expression of selected genes the KFC cells were used. The n = 3 replicates were treated with 1 µM, and 10 µM of zoledronic acid (Sigma) for 24 h. The control cells were treated with medium not containing the ZOL. After treatment, the cells were collected into 1 ml of Tri-Reagent (Sigma) for subsequent RNA extraction.

### Oxysterol Determination During Viral Infections

KFC cells were grown in 27 culture vessels with a surface of 75 cm^2^. 24h post-seeding n = 3 replicates were infected with one of the studied viruses (CyHV-3, IPNV, CSV, CCIV, Para, Ortho, or SVCV) at a MOI of 10 for 24h. Next, the medium was removed, the cells were washed twice with PBS and then scraped off and suspended in 1 ml of PBS. Then, cells were adjusted to 8 × 10^6^ cells per 1 ml of PBS and frozen at −80°C until processing for HPLC-MS. The rest of the culture vessels were used for two sets of controls (n = 3 replicates each) which included mock infected KFC cells cultured in medium with or without FCS.

### Virus Titration

The abundance of infective virus particles was quantified in tissue culture supernatants or infected cells by means of the 50% tissue culture infective dose (TCID_50_) assay, which was performed in 96-well plates according to the method described by Reed and Muench (41). KFC cells were used for titration of CyHV-3, SVCV, and Para. RTG-2 cells were used for titration of VHSV and IPNV. Virus-infected plates were incubated in a humidified atmosphere with 2% CO_2_ at 20°C and monitored daily for the appearance of CPE up to day 7 (SVCV, Para) or day 14 p.i. (VHSV, IPNV, and CyHV-3).

### DNA/RNA Extraction and cDNA Synthesis

DNA was extracted using the QIAamp DNA Mini Kit (QIAGEN, Germany) according to the manufacturer’s instructions. Total RNA was extracted using Tri-Reagent (Sigma) in accordance with the manufacturer’s instructions. Any remaining genomic DNA was digested with 2 U of DNase I (Thermo Fisher Scientific, Germany) according to the manufacturer’s protocol. Synthesis of cDNA was performed from 300 ng of total RNA using the Maxima™ First Strand cDNA Synthesis Kit (Thermo Fisher Scientific). A non-reverse transcriptase control was included in the analysis of each sample. cDNA samples were diluted 1:20 with nuclease-free water prior to RT-qPCR analysis.

### qPCR and RT-qPCR

For quantification of viral gDNA probe based qPCR was used. For quantification of viral and host mRNA and host gDNA a SYBR Green based RT-qPCR was used as described earlier (42). Reactions were performed in duplicate using the Maxima SYBR Green 2× or Maxima Probe Master Mix (Thermo Fisher Scientific) in an ABI StepOnePlus (Thermo Fisher Scientific). The probe reaction mix was prepared as follows: 1× Maxima Probe Master Mix (with 10 nM of ROX), 0.8 μM of each primer, 0.1 μM of the fluorescent probe for CyHV-3, 5 μl of extracted gDNA (in concentration of 50 ng μl) and nuclease-free water to a final volume of 20 μl. The SYBR Green reaction mix was prepared as follows: 1× Maxima SYBR Green Master Mix (with 10 nM of ROX), 0.2 μM of each primer, 5 μl of 20×diluted cDNA or 5 μl of extracted gDNA and nuclease-free water to a final volume of 20 μl. The sequences of the primers are listed in **Table 1**. Due to the fact that fish encode several paralogues of a single CH25H gene in human we provide the localization of the primers for *ch25h_b* of common carp is shown in **Supplementary Figure S1**, the localization of the primers for *ch25h_b* of rainbow trout and Chinook salmon is shown in **Supplementary Figure S2**. A list of all *ch25h* paralogues from common carp, rainbow trout, and Chinook salmon is provided in **Supplementary Table S1**. The phylogenetic analysis and the protein sequence alignments showing that *ch25h_b* of fish is the closest homologue of human single gene CH25H encoding cholesterol 25-hydroxylase are presented in **Supplementary Figures S3** and **S4**.

**Table 1:** Sequences of the PCR primers used in this work.

Gene	Host	Primer/probe sequence	Reference or GenBank ID
*ch25h_b*	Common carp	CCATTCAGAAAACAGAACTGAAAAG	XM_019113007
		TTCATGTAGTTTGTTCCATTTGC	
	Common carp	ATACCCATGTCCTGCCATTC	XM_019113012
		TGTAGTTTGTTCCTGTTGCTCA	
	Common carp	TGGTGCCTTTTGGTCTGT	MT602517/XM_019113007/MT602518/XM_019113012/JZ503992
		TGTCCCAGTGAGTGAAGTATGG	
*cyp7b1*	Common carp	TGTGGGCATCAGTGGGAAAC	MT636087
		TGCATGTTCTTCTCTCCAACCAC	
*fdps*	Common carp	AGGTGTGGTTCAGAGAAATGGAAGA	MT636086
		TGACAAAGGTCTGCGTGCCTA	
*ifna2*	Common carp	GATGAAGGTGCCATTTCCAAG	(43)
		CACTGTCGTTAGGTTCCATTGCTC	
*vig1/*	Common carp	CGCACCAGAGAGCAGAAAG	(43)
*viperin*		CTCAATAGGCAGCACGAAC	
*mx2/*	Common	ATGACCCAGCAGAAGTGGAG	XM_019081222
*mxb-like*	carp	CAGGAACATTGGCAGAGATG	
*40s*	Common carp	CCGTGGGTGACATCGTTACA	(44)
		TCAGGACATTGAACCTCACTGTCT	
*actin*	Common carp	TCACACCACAGCCGAGAG	(43)
*beta*		CAGGGAGGAGGAGGAAGCAG	
*ch25h_b*	Rainbow trout/Chinook salmon	ATCTGGCTGTCTGTGGAGGA	XM_024409452/XM_024425406/XM_021611166/XM_024386464*/XM_021564786*/XM_024387282*/XM_021575763*/XM_024387283*/XM_021575762*/CDQ94151/GBTD01125112/GBTD01125114/CDQ61409/GBTD01125112
		CCTGTCCCAGTGTGTGAAGT	
*ifn1*	Rainbow trout/Chinook salmon	CACGCGAAGTTATTAGCAGTTGAA	(45)
		AAATTATAGTTGAACCACAATGAAATATTATTC	
*mx1-2*	Rainbow trout	GGTTGTGCCATGCAACGTT	(46)
		GGCTTGGTCAGGATGCCTAAT	
*mx1-2*	Chinook salmon	GATGCTGCACCTCAAGTCCTATTA	(47)
		CGGATCACCATGGGAATCTGA	
*irf1*	Chinook salmon	TTCTACACATCTTTCCAAGTGTCA	(30)
		GGGTTTCTTGGTGACTGTCTT	
*cyp7b1*	Rainbow	CTTGCGGCTGGAGGGAGA	XM_021589543/XM_021589542/
	trout	GCTCTGGGGATACAGGGAGA	XM_021613210
*fdps*	Rainbow	TGAGGCCCAGTTTGAGGA	XM_021598291/XM_021598292/
	trout	AGCTCAGTGGGAGGAACAAG	FR946686/FR967682
*vig1/viperin*	Rainbow trout	ACAAAGTGGCGTTCAAAATC	NM_001124253
		ACTGTTCTCCCCAGCGTTC	
*ef1a*	Rainbow trout/Chinook salmon	TGGGCTGGTTCAAGGGATGG	(48)
		CTGGAGGGGCAGACGAAGG	
*beta actin*	Rainbow trout/Chinook salmon	CGCTGGACTTTGAGCAGGAG	AJ438158
		CTCGTTGCCGATGGTGATG	
*DNAh*	CyHV-3	ACGCCTCGCTCAAGAACAC	(43)
		GCCCACCTTCACCACCAC	
*DNAp*	CyHV-3	CGCCGACTTTACCTTTCTGG	(43)
		AACCCTTCCTTGTGCTTGTC	
*mcp*	CyHV-3	AGCCACCTCTTGGTCGTG	(43)
		ACTCCCTGTCCCAGCACTC	
*orf89*	CyHV-3	GACGCCGGAGACCTTGTG	(49)
		CGGGTTCTTATTTTTGTCCTTGTT	
		[FAM]-CTTCCTCTGCTCGGCGAGCACG-[BHQ1]	
*G*	SVCV	GCTACATCGCATTCCTTTTGC	(50)
		GCTGAATTACAGGTTGCCATGAT	
*vp3*	Para	TGGTTGGGATACTGGAGTGG	(51)
		TCCTCTTCCTACGGGGCTTT	
*vp2*	IPNV	CCGCAACTTACTTGAGATCCATTATGC	(52)
		CGTCTGGTTCAGATTCCACCTGTAGTG	
*N*	VHSV	GAATCCGTGCAGCTTTTTCAGG	(53)
		CAAGTGCATCCACGATCACCTTC	
*G rvhsv-tomato*	VSHV	GGGGCCCTCCTTCTACTGGTACTC	FN665788
		CGGAGTCCCGTAATTTGGAAT	

*with one point-mutation in the middle part of one of the primers giving 95% identity.

For quantification of the transcription of rainbow trout genes (designated with the prefix *Onmy*) and Chinook salmon genes (designated with the prefix *Onts*) the delta delta Ct method was used to assure better interchangeability between the laboratories conducting the work on salmonid cells (54). The genes encoding the elongation factor 1 alpha and beta actin of rainbow trout were used as reference genes. Due to the very high similarity of rainbow trout and Chinook salmon and high conservation of the genes used the same primers as in rainbow trout cells were used in Chinook salmon cells after checking correct alignment of the primers to the genome sequence of Chinook salmon. qPCR for the Chinook salmon *mx1-2* and *irf1* genes was carried out as described by Dehler et al. (30).

For the quantification of the transcription of genes of common carp (designated with the prefix *Cyca*) as total copy numbers, a standard curve was prepared using recombinant DNA plasmids from 10^1^ to 10^7^ gene copies and used for quantifying the copy number of the transcribed gene in each sample as described by Adamek et al. (42).

For a normalization of the expression, the genes encoding the 40S ribosomal protein S11 and beta actin of carp were used as reference genes. The level of gene expression is shown as the copy number of the gene normalized against 1 × 10^5^ copies of the 40S ribosomal protein S11 and beta actin (normalized copy number) according to the following formula:

$$\text{Normalized copy number}=\text{mRNA copies per PCR for target gene}/[(\text{mRNA copies per PCR for reference gene}\;1+\text{mRNA copies per PCR for reference gene}\;2)/2\times 10^{5}]$$

For quantification of virus particles by qPCR or RT-qPCR, the results were normalized against host genomic DNA in the case of CyHV-3, or mRNA in the case of SVCV, Para, VHSV, and IHNV. Genome copies of CyHV-3, SVCV, and Para were normalized against 1 × 10^3^ copies of the 40S ribosomal protein gDNA or mRNA, depending on the virus genome nucleic acid. Genome copies of VHSV and IPNV were normalized against 1 × 10^3^ mRNA copies of elongation factor 1 alpha.

### HPLC–MS

High performance liquid chromatography-atmospheric pressure chemical ionization- mass spectrometry (HPLC–APCI–MS) based analyses and quantification of oxysterols was performed as described previously (55). Briefly, the measurements were performed on a Shimadzu Prominence HPLC (Shimadzu) coupled to a triple quad QTrap API 2 000 mass spectrometer equipped with an APCI ion source (AB Sciex, Darmstadt, Germany). Data was evaluated using Analyst 1.4.2 software (AB Sciex, Darmstadt, Germany). For the analysis of cholesterol and oxysterols, 10 μl of the cell culture extracts were separated on a VDSpher PUR C18-H (3 μm, 150 × 2.0 mm) column (VDS optilab, Berlin, Germany). The binary mobile phase consisted of: (A) 8% of 0.1% formic acid in ultrapure water (H_2_Ou) and (B) 92% ACN/MeOH (1:1, v/v). Isocratic elution was performed at a flow rate of 0.2 ml/min and 22°C column temperature. The mass spectrometer was operated in positive mode with selected ion monitoring (SIM) at m/z values of 367.4 [22(S)-hydroxycholesterol, 25-hydroxycholesterol], 369.4 (cholesterol), 383.4 7α,25-dihydroxycholesterol), 385.4 (7β-hydroxycholesterol, 5,6-α-epoxycholesterol and 5,6-β-epoxycholesterol), 392.4 [internal standard, 7β-hydroxycholesterol-(d7)], and 401.4 amu (7-ketocholesterol). Quantitative measurements were obtained through external and internal standard calibration ranging from 10 to 10 000 ng/ml.

### Statistical Analysis

SigmaPlot 12 software (Systat Software) was used for statistical analysis accordingly to the software package manual. The gene expression and TCID_50_ data were transformed using a log10(x) transformation before further statistical analysis to assure normal distribution and equality of variances (56). Significant differences (p ≤ 0.05) between treatments were assessed using a one-way or two-way analysis of variance (ANOVA) with subsequent pairwise multiple comparisons using the Holm-Sidak method, as the most robust approach to the analysis of obtained data available in SigmaPlot 12 software. Data are presented using Prism 7 software (GraphPad Software).

## Results

### Stimulation of KFC Cells With dsRNA and dsDNA

**Figure 1:**
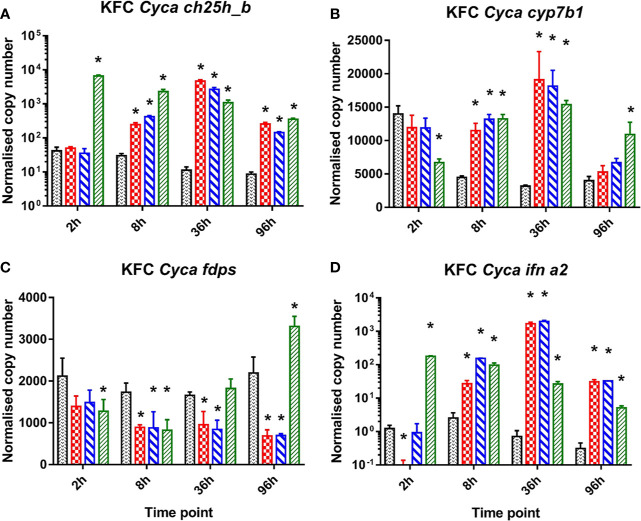
Induction of mRNA encoding enzymes key to oxysterol metabolism and of INFa2 by synthetic analogues to B-form dsDNA, Z-form ds DNA, and dsRNA (poly I:C) as ligand to cytosolic sensors of DNA or dsRNA. Analysis of mRNA encoding for common carp (*Cyca*): **(A)** cholesterol 25 hydroxylase (*ch25h_b*), the enzyme involved in generating 25-hydroxycholesterol from cholesterol, **(B)** cytochrome P450 family 7 subfamily b member 1 *cyp7b1* the enzyme involved in generating 7α,25diHC and **(C)** farnesyl diphosphate synthase (*fdps*), a key enzyme in the sterol pathway, **(D)** IFN a2 (*ifna2*). Data presented as a bar indicating mean normalized copy numbers (+SD) from n = 3 replicates. * indicates the statistically significant difference at p<0.05 between control and stimulated cells. Analysis was performed with two-way ANOVA with treatments and time points as factors. The ANOVA was followed by subsequent pairwise multiple comparisons using the Holm-Sidak method.

In carp cells stimulated with ligands to cytosolic DNA or dsRNA sensors (dsRNA, B-form dsDNA, Z-form dsDNA) of PRR a strong induction of the mRNA expression of *ch25h_b*, the gene encoding the enzyme catalyzing cholesterol to 25-hydroxycholesterol (**Figure 1A**). They also caused a relative upregulation of the *cyp7b1* gene encoding for an enzyme metabolizing 25HC to 7α,25di-hydroxycholesterol (**Figure 1B**) while *fdps*—the gene encoding the enzyme involved in cholesterol synthesis—was downregulated (**Figure 1C**). The cells were also analyzed for an induction of mRNA encoding IFN a2 (*ifna2*) which was significantly upregulated (**Figure 1D**). Interestingly the KFC cells when treated with dsRNA showed different response kinetics compared to when treated with dsDNA. The poly I:C stimulated cells responded immediately with a very rapid increase of *ch25h_b* mRNA already by 2 h post-stimulation (hps) (**Figure 1A**). This was paralleled by an upregulation of *ifna2* (**Figure 1D**) and a downregulation of the transcription of FDPS- encoding mRNA (**Figure 1C**). The cells treated with B-DNA or Z-DNA were responding much slower and obtained the highest transcription rate of *ch25h_b* (**Figure 1A**), *cyp7b1* (**Figure 1B**), and *ifna2* (**Figure 1D**) at later time points (36 hps) of the experiment. At this time point the highest (over 400-fold) upregulation of *ch25h_b* was recorded during Z-DNA stimulation (**Figure 1B**), while *cyp7b1* was upregulated 6-fold in the same samples (**Figure 1D**). Furthermore, over 2 700-fold upregulation of *ifna2* was recorded after B-DNA stimulation (**Figure 1**). Interestingly this shows that the transcription of *ch25h_b* followed the same kinetic as the type I IFN encoding gene after both dsRNA and dsDNA exposure (**Figures 1A, D** **)**.

### Sterol and IFN1 Responses to Viral Infections *In Vitro*

**Figure 2:**
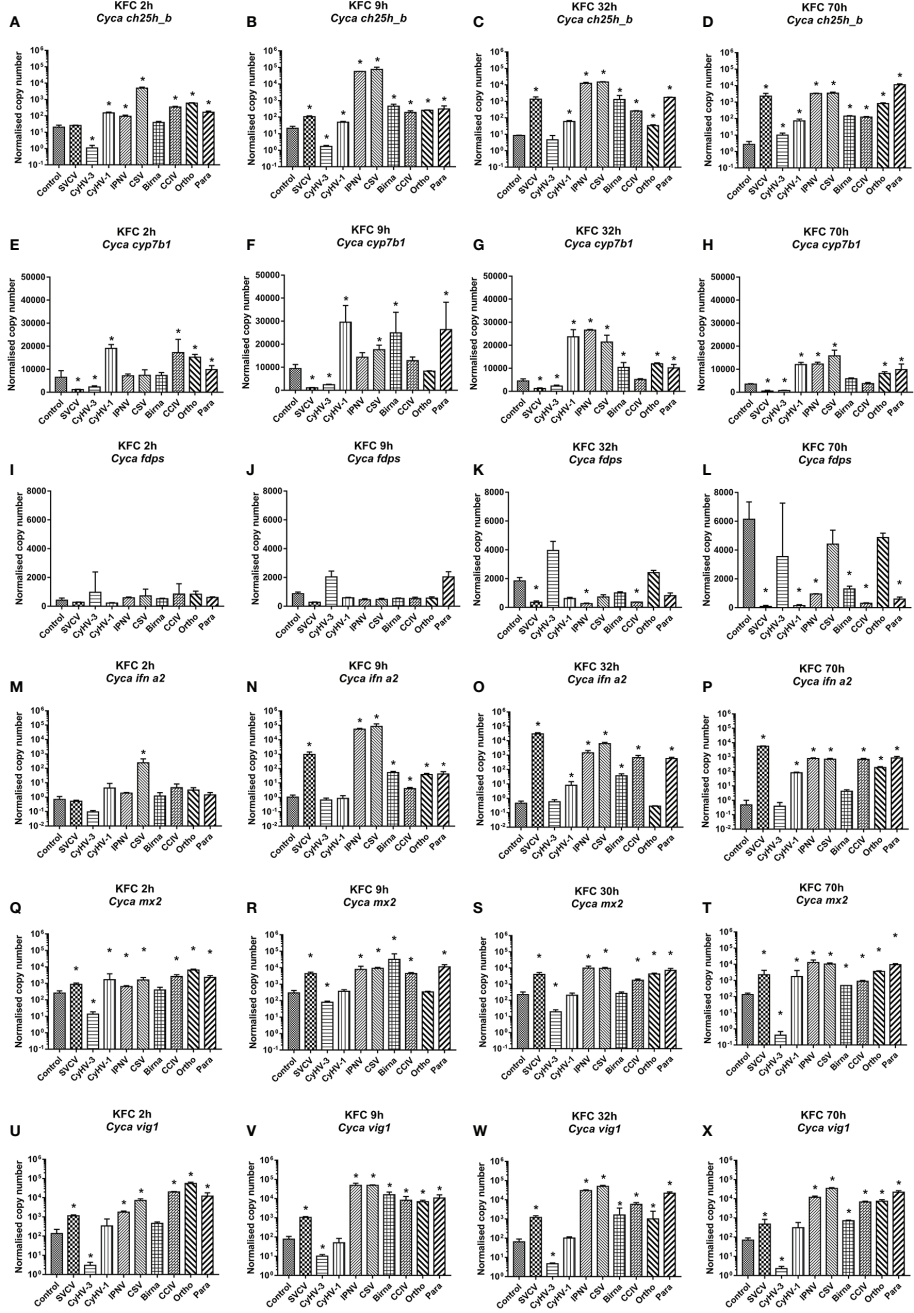
Transcription of mRNA encoding enzymes involved in the generation of oxysterols **(A–D)** *ch25h_b*, **(E–H)** *cyp7b1*, **(I–L)** *fdps* an enzyme in the sterol pathway, **(M–P)** *ifna2*, **(Q–T)** the IFN stimulated gene *mx2* and **(U–X)** *vig1* in carp cells under virus infection at 2, 9, 32, and 70h post-infection. The cells were infected with: *spring viremia of carp virus* (SVCV), *cyprinid herpesvirus 1* (CyHV-1), *cyprinid herpesvirus 3* (CyHV-3), *infectious pancreas necrosis virus* (IPNV), chum salmon reovirus (CSV), an uncharacterized birnavirus isolate infecting carp (Birna), common carp iridovirus (CCIV), an uncharacterized orthomyxovirus infecting carp (Ortho), an uncharacterized paramyxovirus from carp (Para) at MOI of 10. Data presented as a bar indicating mean normalized copy numbers (+SD) from n = 3 replicates. * indicates a statistically significant difference at p<0.05 between control and infected cells. Analysis was performed with two-way ANOVA with infections and time points as factors. The ANOVA was followed by subsequent pairwise multiple comparisons using the Holm-Sidak method.

For a more detailed analysis of *ch25h_b* expression KFC cells were infected with a set of nine different viruses, which replicate in KFC cells. Subsequently, the KFC cells were analyzed for an induction of mRNA transcription of the following genes: *ch25h_b* (**Figures 2A–D**), *cyp7b1* (**Figures 2E–H**)*, fdps* (**Figures 2I–L**), *ifna2* (**Figures 2M–P**), *mx2* (**Figures 2Q–T**), and *vig1* (**Figures 2U–X**). The results showed that *ch25h_b*, *cyp71b*, and *fdps* expression and type I IFN responses are dependent on the type of virus. Furthermore, the level of *ch25h_b* expression (**Figures 2A–D**) again highly reflected the magnitude of *ifna2* (**Figures 2M–P**). The CyHV-3 infection significantly downregulated the expression of *ch25h_b* with up to 20-fold decrease at 2 and 9 hpi (**Figures 2A, B** **)**, the *cyp7b1* (**Figures 2E, F** **)** and the genes of IFN1 response *mx2* and *vig1* (**Figures 2Q–X**) were also downregulated during CyHV-3 infection relative to uninfected control cells. In parallel, in cells under infection with IPNV and CSV, an immediate (by 9 hpi) and strong upregulation of the transcription of *ch25h_b* > 2.700-fold in IPNV and >3 600-fold in CSV infected cells was detected (**Figure 2B**). The *cyp7b1* and genes encoding for type I IFN were also upregulated at the same time point (**Figures 2F, N, V**). The transcription of *ifna2* was upregulated >50 000-fold for IPNV and nearly 80 000-fold under CSV infection (**Figure 2N**), *mx2* was upregulated >70-fold under IPNV and CSV infection (**Figure 2T**), while *vig1* was upregulated >600-fold under IPNV and CSV infection (**Figure 2V**). In cells infected with SVCV, CCIV, or the orthomyxo- and paramyxoviruses from carp an upregulation of the transcription of genes encoding IFN1 response and oxysterol generating enzymes occurred in parallel at later time points e.g., 32 and 70h post-infection (**Figures 2C, D, G, H, O, P, S, T, W, X**). The expression of *fdps* was increasing in the controls during the experiment (**Figures 2I–L**). In comparison to the control, in most of the viral infections the expression was lower with a downregulation of >100 fold. Especially SVCV, IPNV and CCIV caused the highest statistically significant downregulation at 32 and 70 hpi (**Figures 2K, L** **)**. Infection with two viruses CyHV-3 and Para caused (2- to 3-fold) upregulation of *fdps* expression at 9 hpi however, this was not statistically significant (**Figure 2J**).

**Figure 3:**
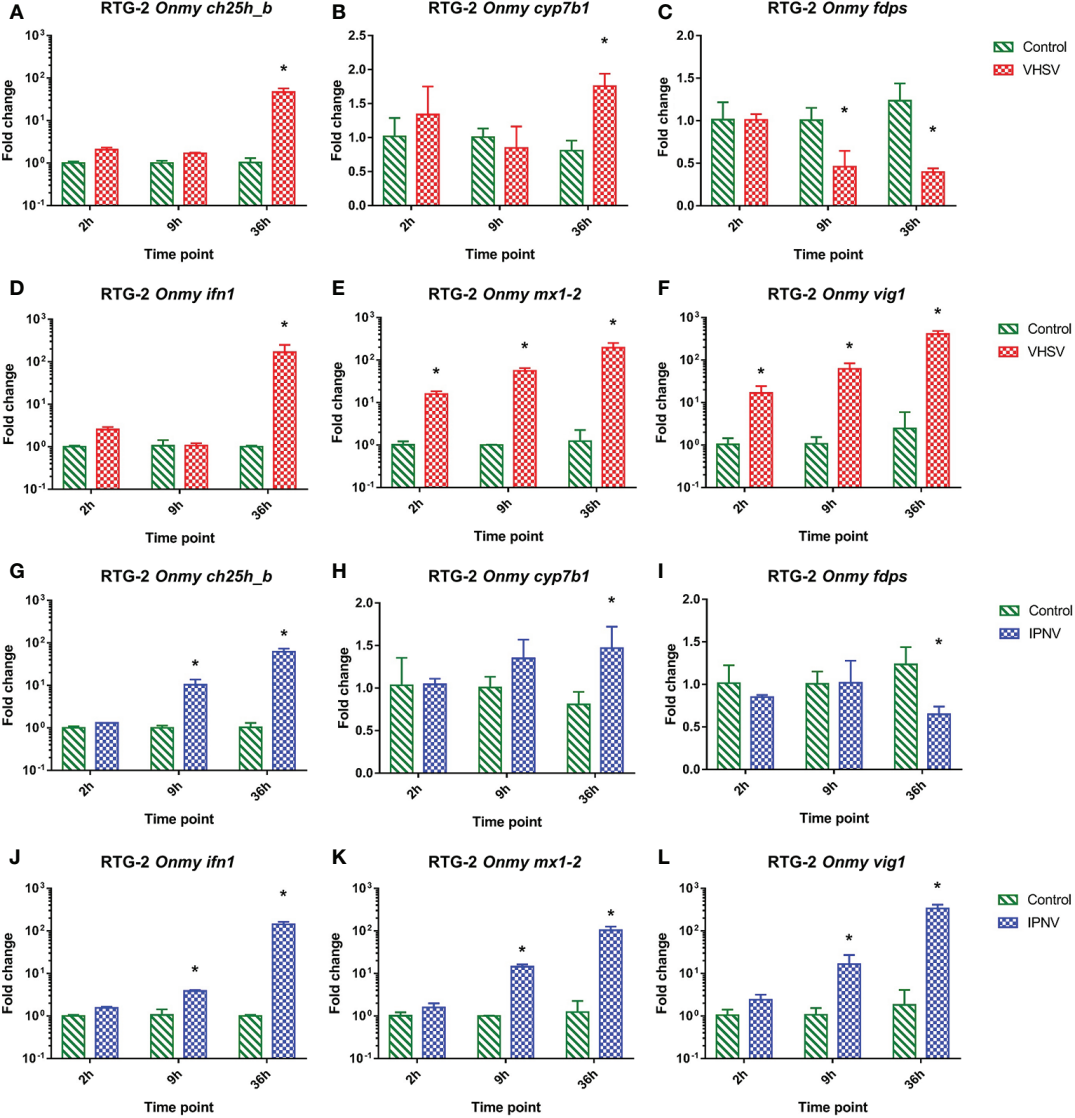
Effect of *viral hemorrhagic septicemia virus* (VHSV) and *infectious pancreatic necrosis virus* (IPNV) infection at MOI of 10 on mRNA levels for rainbow trout (*Onmy*) oxysterol generating enzymes *ch25h_b* **(A, G)**, *cyp7b1* **(B, H)**, and enzyme in the sterol biosynthesis pathway *fdps* **(C, I)**, IFN1 (*ifn a1*) **(D, J)**, the IFN1 stimulated genes *mx1-2* **(E, K)**, and *vig1* **(F, L)**. Data presented as a bar indicating mean fold change (+SD) from n = 3 replicates. * indicates a statistically significant difference at p<0.05 between control and infected cells. Analysis was performed with two-way ANOVA with infections and time points as factors. The ANOVA was followed by subsequent pairwise multiple comparisons using the Holm-Sidak method.

In RTG-2 cells infected with VHSV the mRNA expression of *ch25h_b* (**Figure 3A**), *cyp7b1* (**Figure 3B** **)**, *fdps* (**Figure 3C**), *inf1* (**Figure 3D**), *mx1-2* (**Figure 3E**), and *vig1* (**Figure 3F**) was similarly regulated to the response to IPNV infection (**Figures 3G–L**). They both resembled responses of KFC cells to these infections described above. VHSV and IPNV infections led to a strong upregulation of *ifn1* at 36 hpi (>160-fold in VHSV and >140-fold in IPNV) (**Figures 3D, J** **)**, while *vig1* and *mx1-2* were upregulated as soon as 2 hpi and reached the highest level after 36h p.i. (**Figures 3E, F, K, L** **)**. The rainbow trout *ch25h_b* responded in the same fashion as *ifn1* with an upregulation of the transcription by 46-fold under VHSV infection and by 62-fold under IPNV infection after 36 hpi (**Figures 3A, G** **)**. The expression of *cyp7b1* was significantly upregulated (by 1.5 to 2-fold) at 36 hpi (**Figures 3B, H** **)** while *fdps* was significantly downregulated (by 1.5 to 2-fold) at 9 and 36 hpi during VHSV infection (**Figure 3**) and 36 hpi during IPNV infection (**Figure 3I**).

### Detection of 25HC and 7α,25diHC in Cells Under Virus Infection

**Figure 4:**
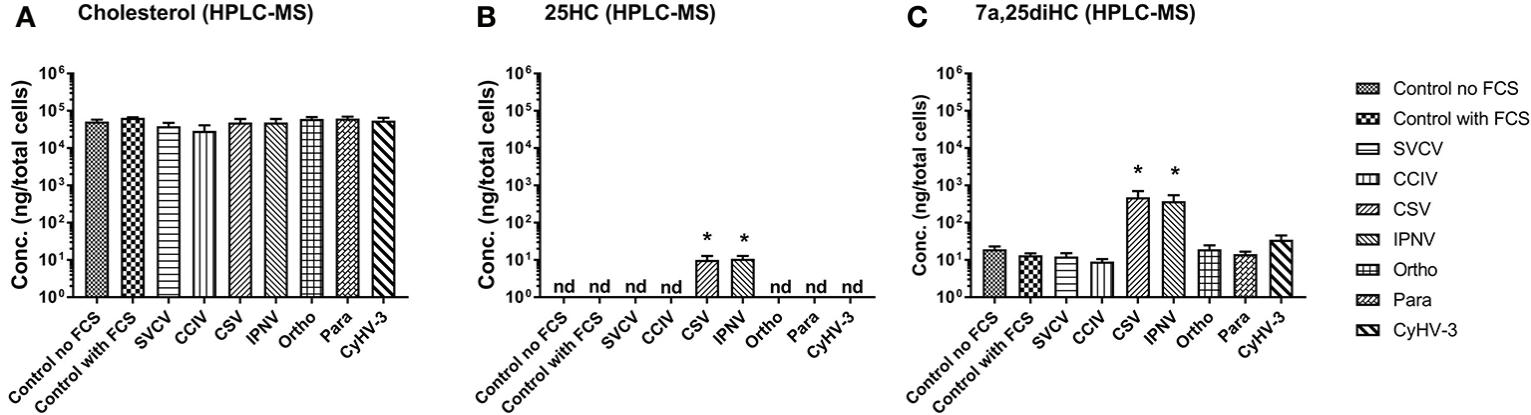
Concentration of **(A)** cholesterol and **(B, C)** oxysterols, **(B)** 25-hydroxycholesterol (25HC), and **(C)** 7α 25hydroxycholesterol (7α,25diHC) in carp cells 24 h post-infection with various viruses replicating in carp cells, as determined by high performance liquid chromatography-atmospheric pressure chemical ionization- mass spectrometry (HPLC-MS). The cells were infected with: *spring viremia of carp virus* (SVCV), *cyprinid herpesvirus 1* (CyHV-1), *cyprinid herpesvirus 3* (CyHV-3), *infectious pancreas necrosis virus* (IPNV), chum salmon reovirus (CSV), an uncharacterized birnavirus isolate infecting carp (Birna), common carp iridovirus (CCIV), an orthomyxovirus infecting carp (Ortho), a paramyxovirus from carp (Para). Data presented as a bar indicating mean concentration (+SD) per 8 x 10^6^ cells from n = 4 replicates. * indicates a statistically significant difference at p<0.05 between control and infected cells. Analysis was performed with one-way ANOVA with subsequent pairwise multiple comparisons using the Holm-Sidak method.

In order to confirm, whether the upregulation of genes encoding oxysterol generating enzymes had an impact on the sterol metabolism of carp cells, the concentration of cholesterol (**Figure 4A**) and the oxysterols 25HC (**Figure 4B**) and 7α,25diHC (**Figure 4C**) were analyzed in KFC cells 24h post-virus infection by means of HPLC-MS using an assay developed by Beck et al. (55). The measurements revealed significantly increased quantities of 25HC and 7α,25diHC in cells under CSV and IPNV-infection (**Figures 4B, C** **)**. In CSV and IPNV infected cells approximately 10 ng of 25HC (per 8 × 10^6^ cells) could be measured. In non-infected cells and in cells under CyHV-1, CyHV-3, Para, Ortho, Birna, and SVCV infections, the amount of 25HC was below the detection limit of the method of 1pg (**Figure 4B**). Also, 7α,25diHC increased during CSV and IPNV infection to 350–500ng (per 8 × 10^6^ cells), while in non-infected cells it was below 20ng (**Figure 4C**). All other infections (CyHV-1, CyHV-3, para, ortho, birna, and SVCV) did lead to a significant increase of 7α,25diHC (**Figure 4C**). Furthermore, no statistically significant alteration of the concentration of cholesterol could be noted in the cells under all virus infections with the employed analytical method (**Figure 4A**).

### Influence of 25HC on Virus Entry, Attachment, and Infection

**Figure 5:**
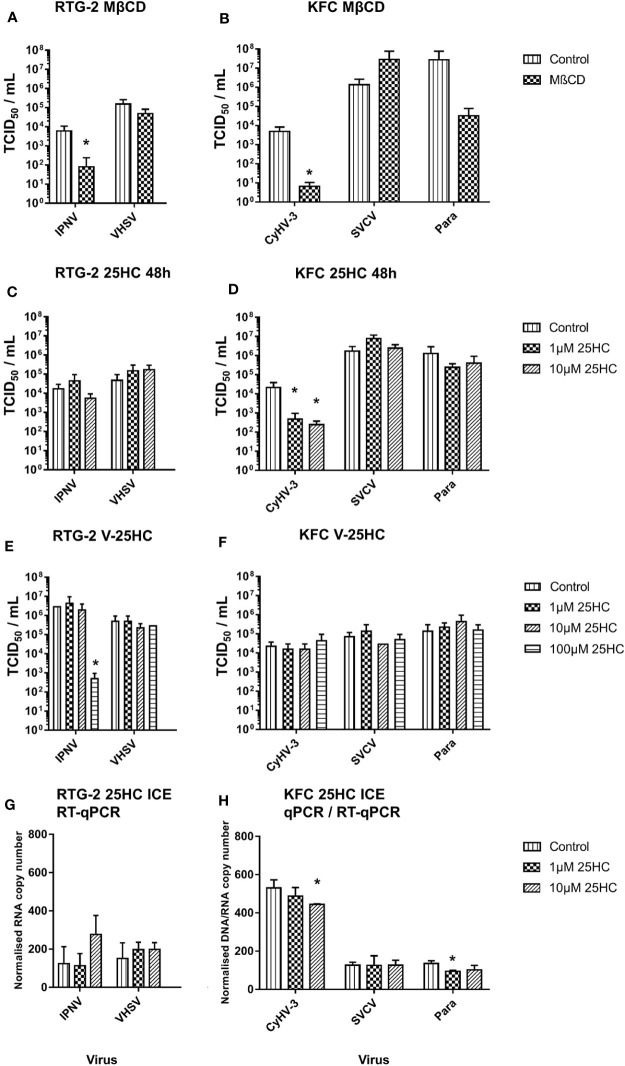
Effect of an incubation of carp cells (KFC) or rainbow trout gonad (RTG-2) cells with **(A, B)** methyl-β-cyclodextrin (MβCD) and **(C, D)** 25-hydroxycholesterol (25HC) prior to infection with CyHV-3, infectious pancreatic necrosis virus (IPNV), a carp pathogenic paramyxovirus (Para), SVCV, or VHSV. **(E, F)** effect of an incubation of the above mentioned viruses with 25HC prior to the infection of the cells on virus replication, monitored by TCID_50_ titering. **(G, H)** Effect of 25HC pre-treatment on attachment of the viruses measured after 2h post-infection on ice using qPCR or RT-qPCR. Data presented as a bar indicating mean TCID_50_/ml or normalized DNA/RNA copy numbers (+SD) from n = 3 replicates. * indicates a statistically significant difference at p < 0.05 between control and the treatments. Analysis was performed with one-way ANOVA with subsequent pairwise multiple comparisons using the Holm-Sidak method.

A reduction of the cholesterol content in the cell membrane of carp and rainbow trout cells by methyl-β-cyclodextrin affected the entry of CyHV-3 and IPNV (>70-fold decrease of IPN virus particles; **Figure 5A**) and CyHV-3 (>700-fold decrease in CyHV-3 virus particles; **Figure 5B**) but not of SVCV, VHSV, or Para (**Figures 5A, B** **)**. A pre-incubation of cells with 1 and 10µM 25HC reduced the entry of CyHV-3 by 46- and 86-fold respectively, but had no effect on the entry of SVCV, the paramyxovirus, IPNV, and VHSV tested (**Figures 5C, D** **)**. A pre-incubation of viruses with 25HC had no influence on the entry of CyHV-3, SVCV, Para, and VHSV, but affected the entry of IPNV and reduced the number of viral particles, which entered the cells by >5 800-fold (**Figures 5E, F** **)**.

In the cells harvested just after infection on ice, the pre-incubation with 10µM 25HC led to reduction of the number of attached CyHV-3 particles by 16%. In the case of Para, pre-incubation with 1µM 25HC decreased the attached viral particle count by 30%. The numbers of VHSV, SVCV, and IPNV particles attached to cells were not affected by 25HC pre-treatment (**Figures 5G, H** **)**.

**Figure 6:**
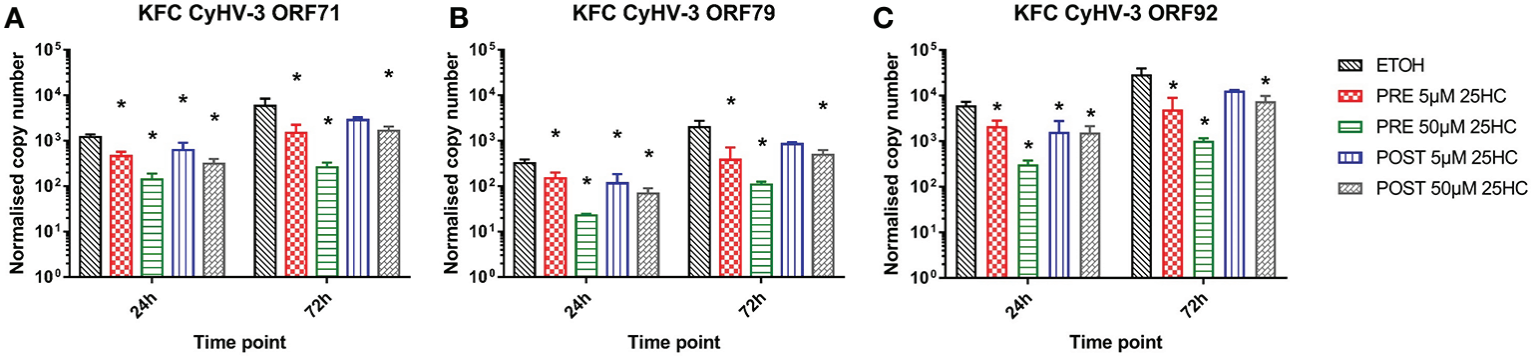
Effect of the incubation of carp cells with 25-hydroxycholesterol (25HC) for 8h prior to (PRE) or 8h post (POST) infection with CyHV-3 on the mRNA transcription of CyHV-3-specific genes **(A)** ORF71, **(B)** ORF79, and **(C)** ORF92 as a surrogate for virus replication. Data presented as a bar indicating mean normalized copy numbers (+SD) from n = 3 replicates. * indicates a statistically significant difference at p<0.05 between control and the treatments. Analysis was performed with two-way ANOVA with treatments and time points as factors. The ANOVA was followed by subsequent pairwise multiple comparisons using the Holm-Sidak method.

In order to further analyze for an antiviral activity of 25HC, we incubated KFC cells with 25HC prior to or after an infection with CyHV-3 (**Figures 6A–C**). The transcription of CyHV-3 specific genes was significantly reduced (up to 7-fold) in a dose-dependent manner, and a pre-incubation of cells with 25HC displayed the highest effect (up to 7-fold reduction in the transcription of CyHV-3-ORF92 after pre-incubation with 50 µM 25HC *vs.* up to 2-fold reduction of the transcription of CyHV-3-ORF71 after incubation with 50 µM 25HC post-infection, **Figures 6A, C** **)**.

The negative influence of 25HC treatment on CyHV-3 entry and replication was confirmed using KF-1 cells. The amount of CyHV-3 DNA was 20-fold and 23-fold lower in medium and cells incubated with 25HC when compared with non-treated control when measured at 4 days post-infection (**Supplementary Figure S5**). The amount of infective virus particles measured by calculating TCID_50_/ml was 162-fold and 383-fold lower in culture medium and cells respectively when 25HC treated cells were compared with the non-treated cells. We summarized the results in **Table 2**.

**Table 2:** Summary of the results presenting the influence of selected viruses on mRNA expression levels of cholesterol 25-hydroxylase and the influence of methyl-β-cyclodextrin (MβCD), 25-hydroxycholesterol (25HC), and zoledronic acid on entry/replication of CyHV-3, SVCV, Para, VHSV, and IPNV

Factor/virus	CyHV-3	SVCV	Para	VHSV	IPNV
**Type I IFN (mRNA expression)**	Downregulation	Upregulation*	Upregulation*	Upregulation*	Upregulation*
**ch25h_b (mRNA expression)**	Downregulation*	Upregulation*	Upregulation*	Upregulation*	Upregulation*
**25HC (oxysterol level)**	No effect	No effect	No effect	No effect	Upregulation*
**7α,25diHC (oxysterol level)**	No effect	No effect	No effect	No effect	Upregulation*
**M**β**CD (entry)**	Blocking*	No effect	No effect	No effect	Blocking*
**25HC (attachment)**	Blocking*	No effect	Blocking	No effect	No effect
**25HC (entry)**	Blocking*	No effect	No effect	No effect	No effect
**25HC (virus incubation-entry)**	No effect	No effect	No effect	No effect	Blocking*
**Zoledronic acid (replication)**	Blocking*	Blocking*	No effect	Not studied	Not studied

*Statistically significant effect at p<0.05. Analyses were performed with one-way or two-way ANOVAs followed by subsequent pairwise multiple comparisons using the Holm-Sidak method.

### Type I IFN Dependence of *ch25h*-Expression

**Figure 7:**
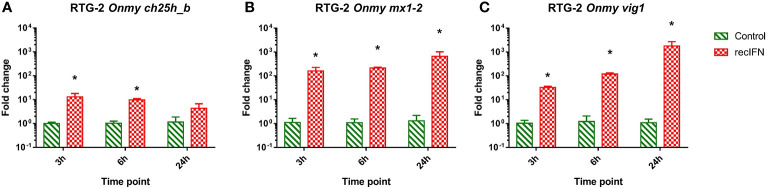
Effect of the incubation of rainbow trout gonad (RTG-2) cells with rainbow trout recombinant IFNa1 (recIFN) on the transcription of mRNA encoding the enzyme involved in the generation of oxysterol *ch25h_b* **(A)**, the IFN stimulated gene *vig1* **(B)**, the IFN stimulated gene *mx1-2* **(C)**. Data presented as a bar indicating mean fold change (+SD) from n = 3 replicates. * indicates a statistically significant difference at p<0.05 between control and the recombinant IFN treatment. Analysis was performed with two-way ANOVA with treatments and time points as factors. The ANOVA was followed by subsequent pairwise multiple comparisons using the Holm-Sidak method.

RTG-2 cells stimulated with rainbow trout rIFNa1 upregulated the mRNA expression of the antiviral genes *mx1-2* (>650-fold after 24h post-stimulation; **Figure 7B**) and *vig1* [>1 750-fold 24 hps.; (**Figure 7C**)] in increasing fashion depending on the time of stimulation with the highest level after 24 hps. In contrast to this, the transcription of *ch25h_b* was upregulated to the highest extent (<10-fold) in the samples from the first time point, 3 hps (**Figure 7A**). A similar dynamic of *ch25h_b* expression was observed during stimulation of KFC cells with rainbow trout rIFNa1, while no upregulation of *ifna2* transcription and the antiviral genes *mx2* and *vig1* was measured (**Supplementary Figure S6**). This indicates that KFC cells were not recognizing rainbow trout rIFNa1, and the *ch25h_b* upregulation in both KFC and RTG-2 cells was not related to rIFNa1 but to possible impurities of the recombinant protein produced in *Escherichia coli*.

**Figure 8:**
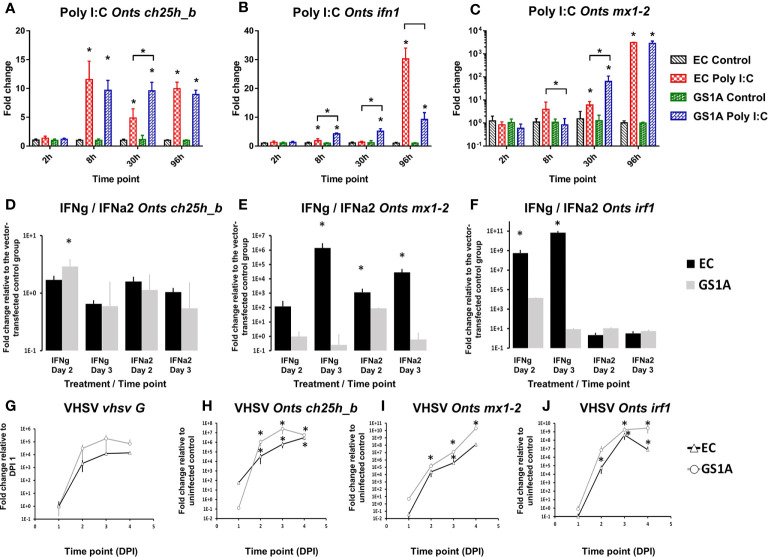
Effect of the stimulation or infection of Chinook salmon cells CHSE-EC and CHSE-GS1A (lacking the ability to produce stat1a1 and stat1a2) on the levels of gene expression. Cells were incubated with poly I:C and the levels of expression of Chinook salmon (*Onts*) *ch25h_b* **(A)**, *mx1-2* **(B)**, *ifn1* **(C)** are presented from n = 5 replicates. Next the cells were transfected with IFNa2 (IFN A2) or IFNγ (IFNG) expression plasmids and the levels of gene expression of *ch25h_b* **(D)** *mx1-2* **(E)** *irf1* **(F)** measured 2 or 3 days post-transfection from n = 3 replicates. This was followed by infection of the cells with rVHSV-tomato (MOI of 1) and the level of expression for *vhsv G* **(G)** *ch25h_b* **(H)** *mx1-2* **(I)** *irf1* **(J)** were measured at 1, 2, 3 or 4 days post-infection from n = 3 replicates. Data presented as a bar or line indicating mean fold change (±SD). * indicates a statistically significant difference at p<0.05 between control and treatment or between different cell lines. Analysis was performed with two-way ANOVA with treatments/cell lines and time points as factors. The ANOVA was followed by subsequent pairwise multiple comparisons using the Holm-Sidak method.

Utilization of CHSE-GS1A cells lacking the ability to produce *stat1a1* and *stat1a2* showed that *ch25h_b* upregulation is not STAT1a1/2 dependent upon poly I:C stimulation. This includes even a statistically higher upregulation of the mRNA expression of *ch25h_b* (**Figure 8A**), IFN a1 (**Figure 8B**), but also the antiviral gene *mx1-2* (**Figure 8C**) at 30 h post-stimulation. The transfection of CHSE-GS1A cells with a plasmid expressing Atlantic salmon IFN γ induced *ch25h_b* significantly at day 2, but only less than 3-fold, and was repressed at day 3 with a fold change of 0.65 (**Figure 8D**) while a plasmid expressing IFN a2 of Atlantic salmon did not induce *ch25h_b* in both cell lines (**Figure 8D**). Concurrently, there was evidence of a potent IFN1 response induced by these plasmids in CHSE-EC cells leading to over 1 000 000-fold upregulation of the antiviral gene *mx1-2* (**Figures 8E, F** **)**, which was clearly impaired in CHSE-GS1A cells where no statistically significant upregulation of this gene could be detected (**Figures 8E, F** **)**.

Using the rVHSV-tomato infection model in the CHSE-EC and CHSE-GS1A, which allows for better monitoring of viral replication, a strong induction of *ch25h_b* in both cell lines was noticed, however in the CHSE-EC cell line the induction was significantly higher (**Figure 8H**). This aligned with the viral replication estimated by the expression level of the G protein which was more pronounced in CHSE-GS1A than in CHSE-EC cells (**Figure 8G**) leading to a generally stronger induction of ISGs (*mx1-2* and *irf1*; **Figures 8I, J** **)**.

### Blocking the FDPS Activity

**Figure 9:**
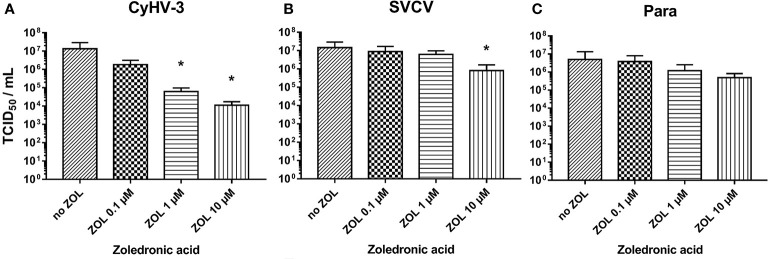
Effect of zoledronic acid (ZOL) treatment on infectivity of **(A)** CyHV-3, **(B)** SVCV, and **(C)** Para. Data presented as a bar indicating mean TCID_50_/ml (+SD) at 7 days post-infection, from n = 6 replicates. * indicates a statistically significant difference at p<0.05 between control and the treatments. Analysis was performed with one-way ANOVA with subsequent pairwise multiple comparisons using the Holm-Sidak method.

The blocking of FDPS activity in KFC-cells by zoledronic acid reduced the replication of CyHV-3. Zoledronic acid (ZOL) showed an extremely strong dose dependent effect on CyHV-3 replication and lowered the virus titer by up to 1 000-fold (**Figure 9A**). Its effect on SVCV and Para replication was noticeably lower (titer reduction by <10-fold) only in the highest dose (**Figures 9B, C** **)**. However, the treatment of cells with zoledronic acid did not have any effect on the expression of genes involved in type I IFN responses or oxysterol synthesis (**Supplementary Figure S7**). The antiviral effect was also not related to cytotoxicity of ZOL. Cytotoxic effect can be only induced in the KFC cells with >100 µM of ZOL, which is a 10 times higher concentration than the highest concentration used in the experiment (data not shown). Furthermore, ZOL did not significantly influence cholesterol levels in cell membranes when compared to MβCD (data not shown).

## Discussion

The entry of the virus into the cell is a critical step in the virus replication cycle, and thus any perturbation of this step can influence the outcome of the infection process. This can, for instance be caused by an incompatibility of the virus to the cellular receptor typically used by the virus to enter the cells, as proposed in the case of a clonal strain of rainbow trout resistant to *viral hemorrhagic septicemia virus* (VHSV) (57). Likewise, the resistance of Atlantic salmon to *infectious pancreatic necrosis virus* (IPNV) was linked to a single nucleotide polymorphism in epithelial cadherin gene *cdh1-1*, which prevented the virus from attaching to and internalizing into cells (58). There is also a growing body of evidence that the modulation of the sterol metabolic network is essential for an antiviral cellular response (8). The products of this network can have direct or indirect antiviral effects and may also disturb the entering process of several mammalian viruses. Recent research has shown that CH25H stimulation *via* interferon stimulated genes (IGSs) results in viral inhibition *via* cell intrinsic and extrinsic mechanisms. This has been shown to have an inhibitory effect on the virus fusion to cell membranes in several enveloped viruses, including herpes simplex virus (59). Additionally in reovirus, a non-enveloped virus, infection, and replication are stunted due to a modulation of viral un-coating in the endosomal compartment (17). Furthermore, an increase in cellular 25HC concentration has been shown to suppress cholesterol biosynthesis in mammalian cells *via* rapid proteasomal elimination of HMG-coenzyme A reductase (60).

Here we present new evidence that the antiviral response based on cholesterol 25-hydroxylase is highly conserved across vertebrates. The upregulation of the gene encoding for cholesterol 25-hydroxylase (in fish *ch25h_b*) could be a part of an antiviral response against a very broad spectrum of viruses in fish cells, derived from both common carp, rainbow trout, and Chinook salmon *in vitro*. Firstly, we showed, that fibroblastic cells are capable of producing 25HC and its metabolite 7α,25diHC under certain infections (e.g., CSV and IPNV). These coincided with an increased transcription of genes encoding proteins from the type I IFN pathway (*ifna2*, *vig1*) and of oxysterol generating enzymes (*ch25h_b, cyp7b1*), and a downregulation of genes encoding for the enzyme responsible for cholesterol synthesis (*fdps*). The detection of 25HC and high levels of 7α,25diHC in CSV and IPNV infected cells could not only be significant for the direct antiviral activity of these cholesterol derivatives, but also could have significant implications for further studies on immunomodulatory activity of oxysterols. 7α,25diHC is a recently recognized ligand for the G protein-coupled receptor GPR183/EBI2, which facilitates chemotaxis of a number of lymphoid cells including B-cells (61).

More importantly, we were able to confirm that 25HC has an antiviral activity by blocking the entry of CyHV-3 virus into cells *in vitro*. Interestingly, from all viruses tested in the present study, CyHV-3 seems to be the only virus highly susceptible to a sequestration or modification of cholesterol in the cell membrane. We were able to influence the replication of CyHV-3 to a high extent by removing cholesterol from the cell membrane using i) MβCD, ii) by blocking the FDPS activity, which is the responsible enzyme for cholesterol synthesis, using zoledronic acid, iii) and finally by 25HC treatment of the cells. Whether this could be related to the replacement of cholesterol in lipid rafts by 25HC, and by this change in the properties of these cholesterol-rich micro domains has to be studied further. In studies on phosphatidylcholine and sphingomyelin synthetic membranes (films), oxysterols including 25HC were able to condense the films, change their in-plane elasticity, and their tendency to form detergent-resistant membrane domains (62). Interestingly, we showed earlier that the lipid composition of the CyHV-3 envelope had some similarities to the lipids present in lipid rafts of carp cell membranes (3). A pre-incubation of CyHV-3 virus with 25HC, however, had no influence on the entry of this virus into carp cells. Further studies should focus on the effect of the lipid micro-domains perturbation (by MβCD or 25HC) on the entry of CyHV-3.

However, the potential susceptibility of CyHV-3 to 25HC based antiviral response seems to be counteracted by this virus action leading to downregulation of the *ch25h_b* gene expression in infected fibroblasts. Likewise, a downregulation of *fdps*, which occurred in cells infected with other viruses like SVCV or IPNV in parallel to the upregulation of *ch25h_b*, was also not present during CyHV-3 infection. The recognition of the virus by cellular receptors should trigger for profound changes in the lipid metabolism of cells (63). Therefore, lack of response could be related to the ability of CyHV-3 to mitigate the activation of this antiviral response in infected cells. Very recent results from RNA-seq of CyHV-3 resistant and susceptible families of common carp putatively indicate that the *ch25h_b* expression levels upon CyHV-3 infection could have an influence on the resistance status (64). However, these results could not be replicated in our *in vivo* studies based on genetically resistant and susceptible strains of common carp infected with CyHV-3 (data not shown) and need to be confirmed by further investigation.

There is a growing amount of research showing the astounding ability of CyHV-3 to circumvent the action of various antiviral immune responses, including type I IFNs, apoptosis, inflammation, or even the development of behavioral fever in infected carp (37, 65–68). These abilities make this virus a master-modulator of carp immune responses. The results of the current study underline that CyHV-3 may require these characteristics to facilitate its ability to infect and replicate in cells. In the current study, a pre-treatment of CyHV-3-susceptible KFC-cells with 25HC reduced the entry and, to some extent, the replication success of CyHV-3.

Evidence grows that in particular large DNA viruses, like herpesviruses, have the ability to take over the cellular lipid metabolism to utilize it for their replication (69). This was confirmed for viruses infecting mammalian cells (69) and as an indication of this process in CyHV-3-infections in carp, could be interpreted the apparent tendency (although not statistically significant) of an upregulation of the transcription of *fdps* at several time-points in the infection process. There are further indications, that changes in the cholesterol synthesis could be a part of the fish cell antiviral response. This was demonstrated by the downregulation of the expression of the *fdps* gene, which encodes for one of the main enzymes in the mevalonate pathway, in the brain of survivors and carriers of IHNV in sockeye salmon. In these fish a prolonged downregulation of the genes encoding for enzymes producing cholesterol was noticed. This suggests that after an infection with a rhabdovirus there is a continued restriction of cholesterol synthesis in fish surviving the infection (14). In our study, the transcription of *fdps* was downregulated in cells treated with ligands to intracellular sensors of DNA or dsRNA PRR, mimicking a virus infection. Interestingly, we also showed that a blocking of FDPS activity with zoledronic acid, which is one of the most potent FDPS blocking drugs (70), could have a strong antiviral effect in the alloherpesviral infection model CyHV-3 but had no or only a minor effect in rhabdoviral infections. Although there is very little information about an antiviral effect of zoledronic acid, other FDPS blocking drugs (like pamidronate) were shown to have such effects (71, 72). The antiviral action of viperin was suggested to be associated with blocking of FDPS, which leads to a perturbation of lipid rafts and subsequently inhibits virus budding from cells, as it was shown, for instance in influenza A virus infections (73). FDPS is also involved in protein prenylation (74), however we were not able to test an antiviral influence of this process.

We tested the influence of oxysterols on the entry of several fish pathogenic viruses *via* pre-incubation of the host cells with 25HC *in vitro*. Although our results did not indicate any anti-SVCV action of 25HC in carp cells, the over-expression of *ch25h_b* transcription had an anti-SVCV effect in zebrafish (16). This may be related to shortcomings in our experimental approach, based on supplementing 25HC in the culture medium rather than overexpressing the hydroxylase gene. Conversely, this may lead to differences in the extent in which divergent entry pathways are affected. The results may also suggest that SVCV and the other viruses tested here, such as common carp paramyxovirus, VHSV, and IPNV are potent inducers of this antiviral response, however are less vulnerable to 25HC. Only a pre-incubation of IPNV, the only non-enveloped virus we studied the entry of, with a high concentration of 25HC decreased the entry of this virus into a host cell, when compared to other viruses from the panel of fish pathogenic viruses. This is an intriguing and puzzling result which needs further investigation into potentially virucidal actions of 25HC against non-enveloped aquatic viruses.

Here, we also give further evidence that despite the fact that a *ch25h_b* based antiviral response coincides with the IFN1 responses, it is not IFN1 dependent, in particular because the transcription of *ch25h_b* did not respond to a stimulation of the cells with recombinant IFN1 and transfection with a plasmid encoding IFN1. In fish cells, an independence of *ch25h_b* activation from the induction of an IFN1 response was first suggested in zebrafish (16). Likewise, in human cells the CH25H response was also found to be IFN1 independent (75) and therefore could not be induced by IFNs. Furthermore, a knockdown of STAT1 expression had no effect on the induction of CH25H. In contrary to this, the activation of CH25H seems to be IFN1 dependent in mice (76) where the sensing of virus or an IFN1 activation induced 25HC synthesis and secretion from the cells. This was facilitated by STAT1 which had the ability to rapidly bind and activate the promoter of the mice *ch25h* (76). Results from the use of *stat1a1/2* knock-out fish cells were less clear. In our study, the *stat1a1* and *stat1a2* lacking cells retained a remarkable ability to mount an antiviral response, when compared to mammalian *stat1* knock-outs, this included an upregulation of mRNA expression of antiviral genes. This suggests that antiviral genes could be upregulated without the involvement of IFN1. In addition, these cells displayed a very similar *ch25h_b* induction, although this reaction occurred earlier than in more classical antiviral IFN1-induced genes.

In conclusion, although in fish cells the expression of *ch25h_b* occurred independently from IFN1, this expression was enhanced concurrently with the induction of IFN1 stimulated genes. The infections inducing the highest upregulation of *ch25h_b* and *cyp7b1* resulted in elevated concentrations of the oxysterols 25HC and 7α,25diHC in fish cells. A pre-treatment of KFC cells with 25HC lowered the entry of CyHV-3 in these cells. In CyHV-3 infected cells, however, an induction of oxysterol generating enzymes as well as increased concentrations of 25HC were not observed. The CyHV-3 infection led to downregulation of *ch25h_b* expression. This further supports the existence of a remarkable ability of CyHV-3 to evade antiviral responses. The strong antiviral effect of 25HC and zoledronic acid against CyHV-3 might have fundamental connotations for further research on innate immune responses focused on influencing the sterol metabolism and formation of lipid rafts in host cells. Furthermore, the cholesterol synthesis pathway could be a suitable target for treatment or prevention of viral infections in fish.

## Data Availability Statement

The original contributions presented in the study are included in the article/**Supplementary Material**. Further inquiries can be directed to the corresponding author.

## Author Contributions

MA performed and analyzed most of the experiments with help from JD and GB. MA, AB, LJ, AMB, and GB analyzed the oxysterol content. LJ and AMB performed additional experiments in KF-1 cells. MM, KR, TR, and BC performed experiments using CHSE-EC and CHSE-GS1A cells. KW, SB, and JZ provided cells, viruses, and recombinant IFN. MA and DS conceived the experimental design of the experiments with contributions from KR, BC, KW, JZ, and SB. MA and DS acquired the funding and wrote the main body of the manuscript with contributions from all other authors. All authors contributed to the article and approved the submitted version.

## Funding

These studies were supported by the Deutsche Forschungsgemeinschaft (DFG project number STE 420/12-1). This publication was supported by Deutsche Forschungsgemeinschaft and University of Veterinary Medicine Hannover, Foundation within the funding program Open Access Publishing.

## Conflict of Interest

The authors declare that the research was conducted in the absence of any commercial or financial relationships that could be construed as a potential conflict of interest.
