# Supplementary material for: Antiviral Actions of 25-Hydroxycholesterol in Fish Vary With the Virus-Host Combination
Source: Front Immunol. 2021 Feb 24;12:581786. doi: 10.3389/fimmu.2021.581786 (PMC7943847; doi:10.3389/fimmu.2021.581786)
Supplement: Supplementary file 1 [file DataSheet_1.docx]

CycaCh25h_MT602517 AGGACCACTGTGGCTATGACCTGCCATGGGCGACACACAGAC**TGGTGCCTTTTGGTCTGT** 660

CycaCh25h_MT602518 AGGACCACTGTGGCTATGACCTGCCATGGGCGACACACAGAC**TGGTGCCTTTTGGTCTGT** 659

************************************************************

CycaCh25h_MT602517 ATGGAGGAGCTCCGCACCATGATGTCCACCATCAGAAGTTCAAGTCCAACTATGCT**CCAT** 720

CycaCh25h_MT602518 ACGGAGGAGCTCCGCACCATGATGTCCACCATCAGAAGTTCAAGTCCAACTATGCT**CCAT** 719

* **********************************************************

CycaCh25h_MT602517 **ACTTCACTCACTGGGACA**AGCTCTTTGGGACACTGCACTCAGAATGAACTG- 771

CycaCh25h_MT602518 **ACTTCACTCACTGGGACA**AGCTCTTTGGGACACTGCACTCTGAATGAACTGA 771

**************************************** **********

**Supplementary Figure S1.** Localisation of the primers used in the qPCR in the common carp (Cyca) *ch25h_b* genes.

Onts_XM_024409452 CCCTAACCTGCTGGGCTGCCACCCGCTCACCAAGATGTTCTTCTTCACCCTGAAC**ATCTG** 1020

Onts_XM_024425406 CCCTAACCTGCTGGGCTGCCACCCGCTCACCAAGATGTTCTTCTTCACCCTGAAC**ATCTG** 738

Onmy_XM_021611166 CCCTAACCTGCTGGGCTGCCACCCGCTCACCAAGATGTTCTTCTTCACCCTGAAC**ATCTG** 621

Onts_XM_024386464 CCCTCTGCTGCTGGGCTGCCACCCCCTGACAGAGATGCTCTTCTATGTACTGAAT**ATCTG** 623

Onmy_XM_021564786 CCCTCTGCTGCTGGGCTGCCACCCCCTGACAGAGATGCTCTTCTATGTACTGAAT**ATCTG** 620

Onts_XM_024387282 CCCTCTGCTGCTGGGCTGCCACCCCCTGACAGAGATGCTCTTCTATGTTCTGAAT**ATCTG** 639

Onmy_XM_021575763 CCCTCTGCTGCTGGGCTGCCACCCCCTGACAGAGATGCTCTTCTATATTCTAAAT**ATCTG** 610

Onts_XM_024387283 CCCTCTGCTGCTGGGCTGCCACCCCCTGACAGAGATGCTCTTCTATGTTCTGAAT**ATCTG** 631

Onmy_XM_021575762 CCCTCTGCTGCTGGGCTGCCACCCCCTGACAGAGATGCTCTTCTATGTTCTGAAT**ATCTG** 627

**** ***************** ** ** ***** ****** ** ** *****

Onts_XM_024409452 **GCTGTCTGTGGAGGA**CCACTCAGGTTATGACCTGCCCTGGGCCCCTCACAGACTGGTACC 1080

Onts_XM_024425406 **GCTGTCTGTGGAGGA**CCACTCAGGTTATGACCTGCCCTGGGCCCCTCACAGACTGGTACC 798

Onmy_XM_021611166 **GCTGTCTGTGGAGGA**CCACTCAGGTTATGACCTGCCCTGGGCCCCTCACAGACTGGTACC 681

Onts_XM_024386464 **GCT**T**TCTGTGGAGGA**CCACTGTGGCTATGACCTGCCCTGGTCCACGCATAGACTGGTGCC 683

Onmy_XM_021564786 **GCT**T**TCTGTGGAGGA**CCACTGTGGCTATGACCTGCCCTGGTCCACGCATAGACTGGTGCC 680

Onts_XM_024387282 **GCT**T**TCTGTGGAGGA**CCACTCTGGCTATGACCTGCCCTGGTCCACGCATAGACTGGTGCC 699

Onmy_XM_021575763 **GCT**T**TCTGTGGAGGA**CCACTCTGGCTATGACCTGCCCTGGTCCACGCATAGACTGGTGCC 670

Onts_XM_024387283 **GCT**T**TCTGTGGAGGA**CCACTCTGGCTATGACCTGCCCTGGTCCACGCATAGACTGGTGCC 691

Onmy_XM_021575762 **GCT**T**TCTGTGGAGGA**CCACTCTGGCTATGACCTGCCCTGGTCCACACATAGACTGGTGCC 687

*** **************** ** *************** ** * ** ******** **

Onts_XM_024409452 CTTTGGGCTCTATGGCGGATCTCCYCACCACGACCTCCACCATCTCAAGTTCATGGTCAA 1140

Onts_XM_024425406 CTTTGGGCTCTATGGCGGATCTCCGCACCACGACCTCCACCATCTCAAGTTCATGGTCAA 858

Onmy_XM_021611166 CTTTGGGCTCTATGGCGGATCTCCACACCACGACCTCCACCATCTCAAGTTCATGGTCAA 741

Onts_XM_024386464 CTTTGGGCTCTACGGTGGAGCTCCGCACCATGACCTGCACCACCTGAAGTTCAAATCCAA 743

Onmy_XM_021564786 CTTTGGACTCTACGGTGGAGCTCCGCACCATGACATGCACCACCTGAAGTTCAAATCCAA 740

Onts_XM_024387282 CTTTGGGCTCTACGGTGGAGCTCCGCACCACGACCTGCACCATCTGAAGTTCAAGTCCAA 759

Onmy_XM_021575763 CTTTGGGCTCTACGGTGGAGCTCCGCACCACGACCTGCACCATCTGAAGTTCAAGTCCAA 730

Onts_XM_024387283 CTTTGGGCTCTACGGTGGAGCTCTGCACCACGACCTGCACCATCTGAAGTTCAAGTCCAA 751

Onmy_XM_021575762 CTTTGGGCTCTACGGTGGAGCTCCGCACCACGACCTGCACCATCTGAAGTTCAAGTCCAA 747

****** ***** ** *** *** ***** *** * ***** ** ******* ***

Onts_XM_024409452 CTACGCACCCT**ACTTCACACACTGGGACAG**GCTGTTCGGCTCGCTGCTGCATACAGACAA 1200

Onts_XM_024425406 CTACGCACCCT**ACTTCACACACTGGGACAG**GCTGTTCGGCTCGCTGCTGCATACAGACAA 918

Onmy_XM_021611166 CTACGCACCCT**ACTTCACACACTGGGACAG**GCTGTTTGGCTCGCTGCTGCATACAGACAA 801

Onts_XM_024386464 CTACGCTCCGT**ACTTCACACACTGGGACAG**GCTTTTTGGGACCTTGCACAAGAGCTCTGA 803

Onmy_XM_021564786 CTACGCTCCGT**ACTTCACACACTGGGACAG**GCTTTTTGGGACCTTGCACAAGAGCTCTGA 800

Onts_XM_024387282 CTATGCTCCGT**ACTTCACACACTGGGACAG**GGTTTTTGGGACATTGCACAAGCATTCAGA 819

Onmy_XM_021575763 CTATGCTCCGT**ACTTCACACACTGGGACAG**GGTTTTTGGGACATTGCACAAGCATTCAGA 790

Onts_XM_024387283 CTATGCTCCGT**ACTTCACACACTGGGACAG**GGTTTTTGGGACATTGCACAAGCATTCAGA 811

Onmy_XM_021575762 CTATGCTCCGT**ACTTCACACACTGGGACAG**GGTTTTTGGGACATTGCACAAGCATTCAGA 807

*** ** ** ********************* * ** ** * *** * *

**Supplementary Figure S2.** Localisation of the primers used in the qPCR in the rainbow trout (Onmy) and Chinook salmon (Onst) *ch25h_b* genes. The point mutation G/T in reverse primers was not present in the TSA and WGS sequences (GenBank ID: CDQ94151, GBTD01125112, GBTD01125114, CDQ61409, GBTD01125112) used for primer design.

**Supplementary Table S1.** GenBank accession numbers for the genes and proteins encoded by the cholesterol 25 hydroxylase paralogues of common carp, rainbow trout and Chinook salmon. Shaded in grey are the entries for homologues of the zebrafish *ch25h_b* gene, which expression was measured. EST - expressed sequence tag, TSA - transcriptome shotgun assembly, WGS - whole genome shotgun.

| **Gene in *Danio rerio* (GenBank ID)** | **Host** | **Nucleotide**  **GenBank ID** | **Protein**  **GenBank ID** | **EST/TSA/WGS**  **GenBank ID** | **Locus** |
| --- | --- | --- | --- | --- | --- |
| *ch25h_a* (MF095413) | Common carp | XM_019097130 | XP_018952675 |  | LOC109082232 |
|  |  | XM_019091588 | XP_018947133 |  | LOC109075736 |
|  | Rainbow trout | XM_021621937 | XP_021477612 |  | LOC110536255 |
|  | Chinook salmon | XM_024442579 | XP_024298347 |  | LOC112265362 |
|  |  | XM_024438376 | XP_024294144 |  | LOC112262684 |
|  |  | XM_024442578 | XP_024298346 |  | LOC112265362 |
|  |  | XM_024438375 | XP_024294143 |  | LOC112262684 |
| *ch25h_b* (MF095414) | Common carp | XM_019113012/MT602518 | XP_018968557 | JZ503992 | LOC109099495 |
|  |  | XM_019113007/MT602517 | XP_018968552 |  | LOC109099486 |
|  | Rainbow trout | XM_021611166 | XP_021466841 | CDQ94151 | LOC110529015 |
|  |  | XM_021575763 | XP_021431438 | GBTD01125112 | LOC110498991 |
|  |  | XM_021564786 | XP_021420461 | GBTD01125114/ CDQ61409 | LOC110491381 |
|  |  | XM_021575762 | XP_021431437 | GBTD01125112 | LOC110498990 |
|  | Chinook | XM_024425406 | XP_024281174 |  | LOC112253430 |
|  | salmon | XM_024409452 | XP_024265220 |  | LOC112241335 |
|  |  | XM_024386464 | XP_024242232 |  | LOC112223449 |
|  |  | XM_024387283 | XP_024243051 |  | LOC112223991 |
|  |  | XM_024387282 | XP_024243050 |  | LOC112223989 |
| *ch25h_c1* | Rainbow trout | XM_021571258 | XP_021426933 |  | LOC110495809 |
| (MF095415) | Chinook salmon | XM_024379494 | XP_024235262 |  | LOC112218585 |
| *ch25h_c2* | Common carp | XM_019122127 | XP_018977672 |  | LOC109109020 |
| (MF095416) | Chinook salmon | XM_024380417 | XP_024236185 |  | LOC112219235 |
| *ch25h_d* (MF095417) | Common carp | XM_019073746 | XP_018929291 |  | LOC109056552 |
|  |  | XM_019071129 | XP_018926674 |  | LOC109053824 |
|  |  | XM_019080025 | XP_018935570 |  | LOC109062966 |
|  | Rainbow trout | XM_021601299 | XP_021456974 |  | LOC110522829 |
|  |  | XM_021590696 | XP_021446371 |  | LOC110509673 |
|  | Chinook salmon | XM_024396823 | XP_024252591 |  | LOC112230551 |

**
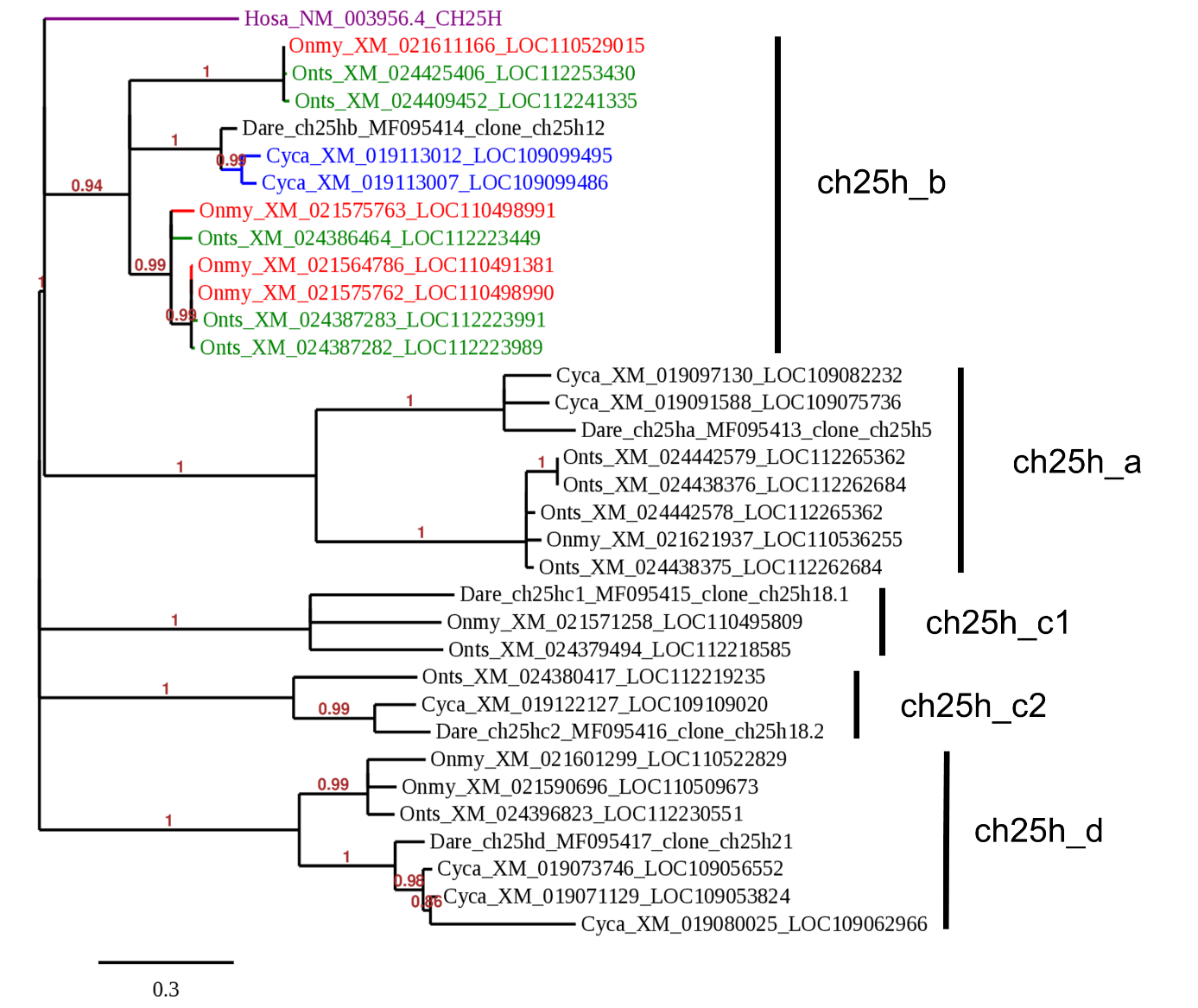
**

**Supplementary Figure S3.** Phylogenetic relation of common carp, rainbow trout and Chinook salmon *ch25h* genes in comparison to human CH25H and zebrafish *ch25h a*, *b*, *c1*, *c2* and *d* genes. A maximum likelihood phylogenetic analysis was performed with PhyML on the coding nucleotide sequence of each gene. The primers used for gene expression were able to amplify homologues of the *danio rerio* *ch25h_b* gene from common carp (Cyca) marked in blue, rainbow trout (Onmy) marked in red and chinnok salmon (Onts) marked in green. Human *CH25H* gene is marked in violet. The phylogenetic tree was rendered with TreeDyn. The phylogenetic analysis was performed using tools available at www.phylogeny.fr. The branch length is proportional to the number of substitutions per site. The branch supporting values are indicated in brown.

HosaCH25H_NP_003947 MSCHNCSDPQVLCSSGQLFLQPLWDHLRSWEALLQSPFFPVIFSITTYVGFCLPFVVLDI 60

Cyca_MT602517 ----------------MFGLQYIWDSILQYEAMLRSPYFPVLFSITVYLSFCLPFVILDV 44

Cyca_MT602518 ----------------MFGLQYIWDSILQYEAVLRSPYFPVFFSITVYLSFCLPFVALDA 44

Onmy_XP_021466841 -------MEPSLESSSPFLLQTLWDKIRAQEDFLRSPLFPVLFSMTLYLSCCLPYLCLDT 53

Onts_XP_024281174 -------MEPSLESSSPFLLQTLWDKIRAQEDFLRSPLFPVLFSMTLYLSCCLPYLCLDT 53

Onts_XP_024265220 -------MEPSLESSSPFLLQTLWDKIRAQEDFLRSPLFPVLFSMTLYLSCCLPYLCLDT 53

Onmy_XP_021420461 -----------------MLLQSLWDLILGYNAWLMSPFFPVLFSLSVYLAFCLPFVVLDL 43

Onts_XP_024242232 -----------------MLLQSLWDLILGYNAWLMSPFFPVLFSLSVYLAFCLPFVVLDL 43

Onts_XP_024243051 -----------------MLPQSLWDFILGYNAWLRSPFFPVLFSLSIYLTFCLPFVVLDL 43

Onmy_XP_021431438 -----------------MLLQSLWDFILGYHAWLRSPFFPVLFSLSVYLTFCLPFVVLDL 43

Onmy_XP_021431437 -----------------MLPQSLWDFILGYHAWLRSPFFPVLFSLSVYLTFCLPFVVLDL 43

Onts_XP_024243050 -----------------MLLQSLWDFILGHHVWLRSPFFPVLFSLSVYLTFCLPFVVLDL 43

: * :** : . * ** ***:**:: *: ***:: **

HosaCH25H_NP_003947 LCSWVPALRRYKIHPDFSPSAQQLLPCLGQTLYQHVMFVFPVTLLHWARSPALLPHEAPE 120

Cyca_MT602517 LSPRVALIRRYKIQQKTSVSWTMMWSCLALSLYNHAMYIFPLSVLHWYWRPVSYPAMAPG 104

Cyca_MT602518 LSSRVSWIRRYKIQQKTSVSWKMMWSCLALSLYNHAVYIFPLSVLHWYWRPVSYPVMAPG 104

Onmy_XP_021466841 LSSRVALVHRYKIQSQSRVTWAMAWSCLATSLHTHAVFIFPLSVLHWYWRPVVLPAQAPG 113

Onts_XP_024281174 LSSRVALVHRYKIQSQSRVTWAMAWSCLATSLHTHAVFIFPLSVLHWYWRPVVLPAQAPG 113

Onts_XP_024265220 LSSRVALVHRYKIQSQSRVTWAMAWSCXATSLHTHAVFIFPLSVLHWYWRPVVLPAXAPG 113

Onmy_XP_021420461 LSPRLAWIRTFKIQQKSHVSWTMMWSCLAHSLYNHVVFLFPLTVLHWYWRPASFIAEAPG 103

Onts_XP_024242232 LSPRLAWIRTFKIQQKSHVSWTMMWSCLAHSFYNHVVFLFPLTVLHWYWRPASFIAEAPG 103

Onts_XP_024243051 LSPRLAWIRTFKIQQKSHVSWTMMWSCLAHSLYNHVVFIFPLTVLHWFWRPATFMPEAPG 103

Onmy_XP_021431438 LSPRLAWIRTFKIQQKSHVSWTMMWSCLAHSLYNHVVFLFPLTVLHWFWRPATFMPEAPG 103

Onmy_XP_021431437 LSPRLAWIRTFKIQQKSHVSWTMMWSCLAHSLYNHVVFLFPLTVLHWFWRPATFMPEAPG 103

Onts_XP_024243050 LSPRLAWIRTFKIQQKSHVSWTMMWSCLAHSLYNHVVFLFPLTVLHWFWRPATFMPEAPG 103

*. : :: :**: . : * . ::: *.:::**:::*** *. **

HosaCH25H_NP_003947 LLLLLHHILFCLLLFDMEFFVW**HLLHH**KVPWLYRTF**HKVHH**QNSSSFALATQYMSVWELF 180

Cyca_MT602517 LLRVIWDLAACLLLFDFQYFVW**HLLHH**KVPWLYRTF**HKVHH**KYTSTFALATEYSGAWEIL 164

Cyca_MT602518 LLRVIWDLAACLLLFDFQYFVW**HLLHH**KVPWLYRTF**HKVHH**KYTSTFALATEYSGAWETL 164

Onmy_XP_021466841 SLRVAWDVLACLLLFDLQYFVW**HVLHH**KVPWLYRTF**HKVHH**RYTATFALTTEHSGIWETL 173

Onts_XP_024281174 SLRVAWDVLACLLLFDLQYFVW**HVLHH**KVPWLYRTF**HKVHH**RYTATFALTTEHSGIWETL 173

Onts_XP_024265220 SLRVAWDVLACLLLFDLQYFVW**HVLHH**KVPWLYRTF**HKVHH**RYTATFALTTEHSGIWETL 173

Onmy_XP_021420461 TLRLIWDVVACLLLFDFQSFIW**HMLHH**KVPWLYRTF**HKVHH**MHTTTFALTTEYSGAWETL 163

Onts_XP_024242232 TLRLIWDVVACLLLFDFQSFIW**HMLHH**KVPWLYRTF**HKVHH**MHTTTFALTTEYSGAWETL 163

Onts_XP_024243051 TLRLIWDVVACLLLFDFQYFIW**HLLHH**KVPWLYRTF**HKVHH**KHTSTFALTTEYSGAWETL 163

Onmy_XP_021431438 TLRLIWDVVACLLLFDFQYFIW**HLLHH**KVPWLYRTF**HKVHH**KHTSTFALTTEYSGAWETL 163

Onmy_XP_021431437 TLRLIWDVVACLLLFDFQYFIW**HLLHH**KVPWLYRTF**HKVHH**KHTSTFALTTEYSGAWETL 163

Onts_XP_024243050 TLRLIWDVVACLLLFDFQYFIW**HLLHH**KVPWLYRTF**HKVHH**KHTSTFALTTEYSGAWETL 163

* : .: ******:: *:**:***************** :::***:*:: . ** :

HosaCH25H_NP_003947 SLGFFDMMNVTLLGCHPLTTLTFHVVNIWLSVEDHSGYNFPWSTHRLVPFGWYGGVVH**HD** 240

Cyca_MT602517 SLGFFAAVNPMLLGVHPMTEMLFHMLNMWLSVEDHCGYDLPWATHRLVPFGLYGGAPH**HD** 224

Cyca_MT602518 SLGFFAAVNPMLLGVHPMTEMLFHILNMWLSVEDHCGYDLPWATHRLVPFGLYGGAPH**HD** 224

Onmy_XP_021466841 SLGLFAAVNPNLLGCHPLTKMFFFTLNIWLSVEDHSGYDLPWAPHRLVPFGLYGGSPH**HD** 233

Onts_XP_024281174 SLGLFAAVNPNLLGCHPLTKMFFFTLNIWLSVEDHSGYDLPWAPHRLVPFGLYGGSPH**HD** 233

Onts_XP_024265220 SLGLFAAVNPNLLGCHPLTKMFFFTLNIWLSVEDHSGYDLPWAPHRLVPFGLYGGSPH**HD** 233

Onmy_XP_021420461 SLGFFSSVNPLLLGCHPLTEMLFYVLNIWLSVEDHCGYDLPWSTHRLVPFGLYGGAPH**HD** 223

Onts_XP_024242232 SLGFFSSVNPLLLGCHPLTEMLFYVLNIWLSVEDHCGYDLPWSTHRLVPFGLYGGAPH**HD** 223

Onts_XP_024243051 SLGFFAGVNPLLLGCHPLTEMLFYVLNIWLSVEDHSGYDLPWSTHRLVPFGLYGGALH**HD** 223

Onmy_XP_021431438 SLGFFAGVNPLLLGCHPLTEMLFYILNIWLSVEDHSGYDLPWSTHRLVPFGLYGGAPH**HD** 223

Onmy_XP_021431437 SLGFFAGVNPLLLGCHPLTEMLFYVLNIWLSVEDHSGYDLPWSTHRLVPFGLYGGAPH**HD** 223

Onts_XP_024243050 SLGFFAGVNPLLLGCHPLTEMLFYVLNIWLSVEDHSGYDLPWSTHRLVPFGLYGGAPH**HD** 223

***:* :* *** **:* : *. :*:*******.**::**: ******* *** ***

HosaCH25H_NP_003947 **LHH**SHFNCNFAPYFTHWDKILGTLRTASVPAR---------------------------- 272

Cyca_MT602517 **VHH**Q*KFKSN*YAPYFTHWDKLFGTLHSE--------------------------------- 251

Cyca_MT602518 **VHH**Q*KFKSN*YAPYFTHWDKLFGTLHSE--------------------------------- 251

Onmy_XP_021466841 **LHH**LKFMVNYAPYFTHWDRLFGSLLHTDKPDTFDVDVLDASKRCDTASSGVTDVSHAPVY 293

Onts_XP_024281174 **LHH**LKFMVNYAPYFTHWDRLFGSLLHTDKPDTFDVDVLDTSKRCDTASSGVTDVSHAPVY 293

Onts_XP_024265220 **LHH**LKFMVNYAPYFTHWDRLFGSLLHTDKPDTFDVDVLDXSKRCDTASSGVTDVSHAPVY 293

Onmy_XP_021420461 **MHH**L*KFKSN*YAPYFTHWDRLFGTLHKSSD------------------------------- 252

Onts_XP_024242232 **LHH**L*KFKSN*YAPYFTHWDRLFGTLHKSSD------------------------------- 252

Onts_XP_024243051 **LHH**L*KFKSN*YAPYFTHWDRVFGTLHKHSD------------------------------- 252

Onmy_XP_021431438 **LHH**L*KFKSN*YAPYFTHWDRVFGTLHKHSD------------------------------- 252

Onmy_XP_021431437 **LHH**L*KFKSN*YAPYFTHWDRVFGTLHKHSD------------------------------- 252

Onts_XP_024243050 **LHH**L*KFKSN*YAPYFTHWDRVFGTLHKHSD------------------------------- 252

:** :* *:********:::*:*

HosaCH25H_NP_003947 ----------------- 272

Cyca_MT602517 ----------------- 251

Cyca_MT602518 ----------------- 251

Onmy_XP_021466841 ETTQSCVKDYEVQFRGK 310

Onts_XP_024281174 ETTQSCVKDYEVQFRGK 310

Onts_XP_024265220 ETTQSCVKDYEVQFRGK 310

Onmy_XP_021420461 ----------------- 252

Onts_XP_024242232 ----------------- 252

Onts_XP_024243051 ----------------- 252

Onmy_XP_021431438 ----------------- 252

Onmy_XP_021431437 ----------------- 252

Onts_XP_024243050 ----------------- 252

**Supplementary Figure S4.** CLUSTAL Omega (1.2.4) multiple amino acid sequence alignment of cholesterol 25 hydroxylase of human (HosaCH25H GenBank ID: NP_003947); and cholesterol 25 hydroxylase b of Chinook salmon (ch25h_b, Onts GenBank ID: Onts_XP_024281174; Onts_XP_024265220; Onts_XP_024242232; Onts_XP_024243051; Onts_XP_024243050); rainbow trout (Onmy GenBank ID: Onmy_XP_021466841; Onmy_XP_021420461; Onmy_XP_021431438; Onmy_XP_021431437) and common carp (Cyca GenBank ID: MT602517; MT602518). The fatty acid hydroxylase superfamily conserved domains are shaded in gray. The histidine cluster motif (HXXHH) characteristic for di-iron-oxo proteins (Fe-O-Fe) such as fatty acid desaturases/hydroxylase enzymes is underlined. This motif is involved in coordination of the reception of electrons in reduction reactions. Putative dilysine motifs (KXKXX, at the C-terminus), thought to signal ER retention, are marked in cursive and underlined. The alignment was performed with the online CLUSTAL Omega tool (https://www.ebi.ac.uk/Tools/msa/clustalo/). Symbols (* : .) under the alignment indicate levels of similarity of amino acids.


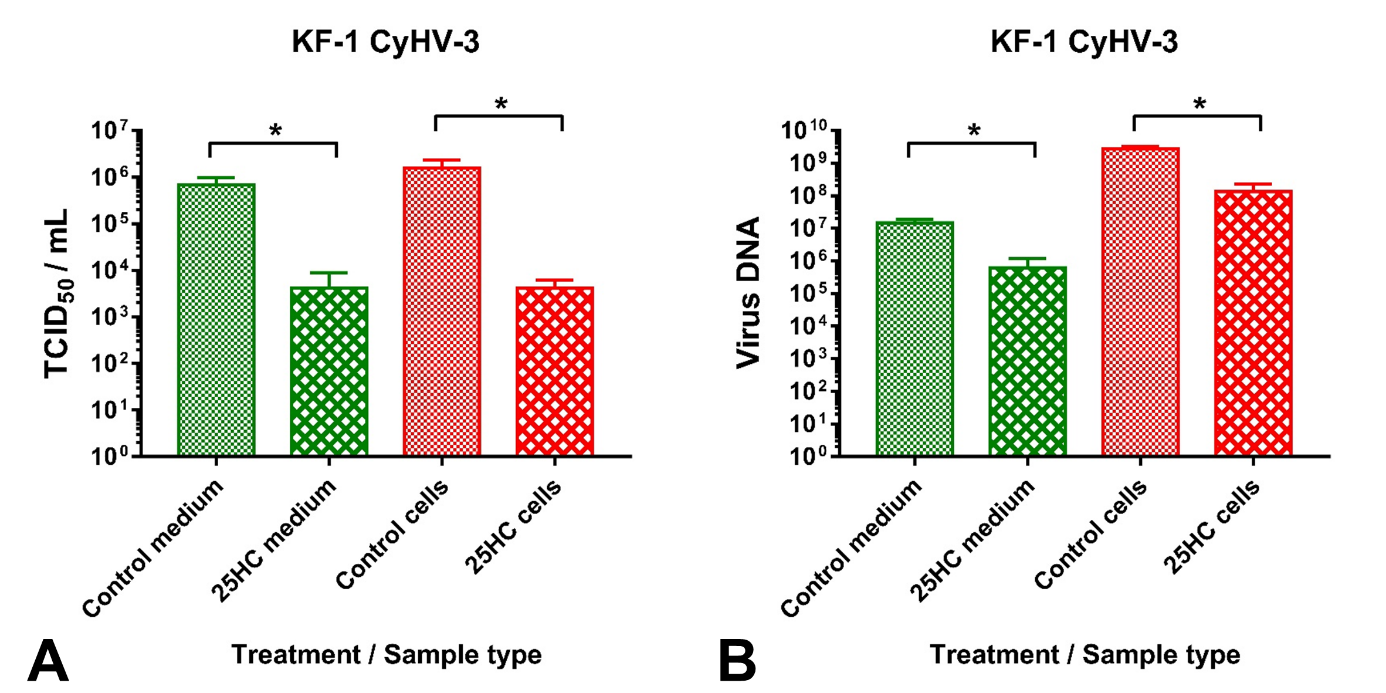


**Supplementary Figure S5.** Effect of the incubation of koi fin 1 (KF-1) cells with 10 µM of 25-hydroxycholesterol (25HC) for 8h prior to infection with CyHV-3 with MOI of 0.001 for 1h, following inoculation in medium with 10 µM of 25HC. Cells were harvested following 96h incubation. Virus content was measured by estimation of TCID_50_ / mL(A) or virus DNA copy numbers with qPCR (B) in medium and cells. Data presented as a bar indicating mean value for TCID_50_ /mL or number of virus DNA copies (+SD). * indicates a statistically significant difference at p<0.05 between control and 25HC pre-treatment. Analysis was performed with two-way ANOVA with subsequent pairwise multiple comparisons using the Holm-Sidak method.


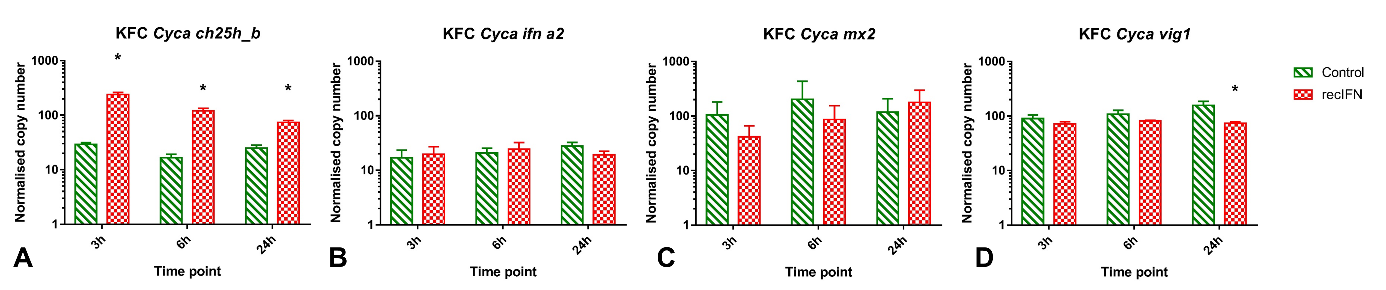


**Supplementary Figure S6.** Effect of incubation of koi fin cells (KFC) with rainbow trout recombinant IFNa1 (recIFN) on the transcription of mRNA encoding the enzyme involved in the generation of 25-hydroxycholesterol *ch25h_b* (A), the IFN gene *ifna2* (B) and the IFN stimulated genes mx2 (C) and *vig1* (D). Data presented as a bar indicating mean normalised copy numbers (+SD) from n=3 replicates. * indicates a statistically significant difference at p<0.05 between control and the recombinant IFN treatment. Analysis was performed with two-way ANOVA with subsequent pairwise multiple comparisons using the Holm-Sidak method.


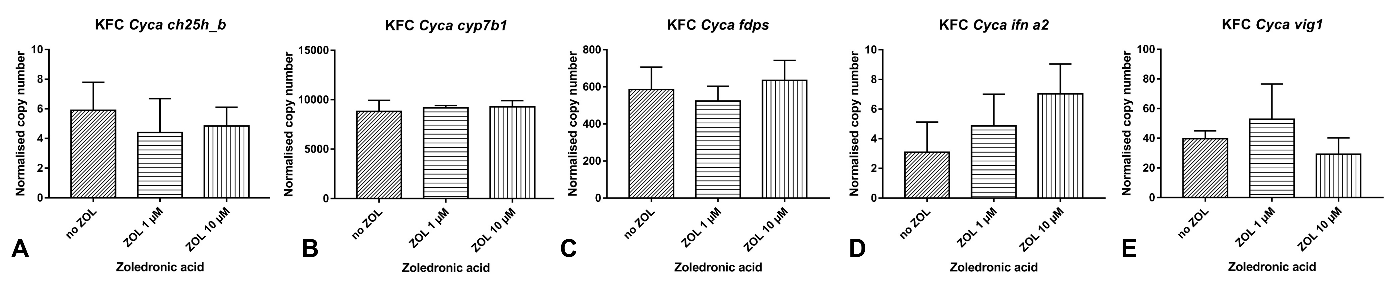


**Supplementary Figure S7.** Effect of 24h incubation of koi fin cells (KFC) with zoledronic acid on the transcription of mRNA encoding the enzyme involved in the generation of oxysterols *ch25h_b* (A) *cyp71b* (B), farnesyl diphosphate synthase (*fdps*), a key enzyme in the sterol pathway(C), the IFN gene *ifna2* (D) and the IFN stimulated gene *vig1* (E). Data presented as a bar indicating mean normalised copy numbers (+SD) from n=3 replicates. No statistically significant difference at p<0.05 between control and treated cells was recorded. Analysis was performed with one-way ANOVA with subsequent pairwise multiple comparisons using the Holm-Sidak method.
